# Bayesian identification of differentially expressed isoforms using a novel joint model of RNA-seq data

**DOI:** 10.1371/journal.pcbi.1012750

**Published:** 2025-01-31

**Authors:** Xu Shi, Xiao Wang, Lu Jin, Leena Halakivi-Clarke, Robert Clarke, Andrew F. Neuwald, Jianhua Xuan

**Affiliations:** 1 Bradley Department of Electrical and Computer Engineering, Virginia Polytechnic Institute and State University, Arlington, Virginia, United Sates of America; 2 Hormel Institute, University of Minnesota, Austin, Minnesota, United Sates of America; 3 Institute for Genome Sciences and Department Biochemistry & Molecular Biology, University of Maryland School of Medicine, Baltimore, Maryland, United Sates of America; University of California Riverside, UNITED STATES OF AMERICA

## Abstract

We develop a Bayesian approach, BayesIso, to identify differentially expressed isoforms from RNA-seq data. The approach features a novel joint model of the sample variability and the deferential state of isoforms. Specifically, the within-sample variability and the between-sample variability of each isoform are modeled by a Poisson-Lognormal model and a Gamma-Gamma model, respectively. Using a Bayesian framework, the differential state of each isoform and the model parameters are jointly estimated by a Markov Chain Monte Carlo (MCMC) method. Extensive studies using simulation and real data demonstrate that BayesIso can effectively detect isoforms of less differentially expressed and differential transcripts for genes with multiple isoforms. We applied the approach to breast cancer RNA-seq data and uncovered a unique set of isoforms that form key pathways associated with breast cancer recurrence. First, PI3K/AKT/mTOR signaling and PTEN signaling pathways are identified as being involved in breast cancer development. Further integrated with protein-protein interaction data, pathways of Jak-STAT, mTOR, MAPK and Wnt signaling are revealed in association with breast cancer recurrence. Finally, several pathways are activated in the early recurrence of breast cancer. In tumors that occur early, members of pathways of cellular metabolism and cell cycle (such as CD36 and TOP2A) are upregulated, while immune response genes such as NFATC1 are downregulated.

## Author summary

A Bayesian approach, BayesIso, is developed to identify differentially expressed isoforms from RNA-seq data. The approach features a novel joint model of the sample variability and the deferential state of isoforms; the deferential state can be estimated by a Markov Chain Monte Carlo (MCMC) method. Extensive studies using simulation and real data demonstrate that BayesIso can effectively detect isoforms of less differentially expressed and differential transcripts for genes with multiple isoforms. We applied the approach to breast cancer RNA-seq data and uncovered differential isoforms that form key pathways associated with breast cancer recurrence.

## 1. Introduction

RNA sequencing (RNA-seq) [1–3] is an important technique for transcriptome analysis of cancer cells. It allows exploration of the transcriptome at the resolution of individual bases, and, with millions of transcript reads, can quantify gene expression with high accuracy. As the cost continues to decrease, more tumor samples will likely be profiled by RNA-seq than by other current techniques.

A key aspect of cancer research is the detection of differentially expressed transcripts (or isoforms) among different types or sub-types of cancer cells [4–6]. While RNA-seq has the advantage of wide coverage and high resolution, many challenges remain for transcriptome analysis, such as the uncertainty of read assignments and the variability of RNA-seq data. For example, because genes often express multiple transcripts (isoforms), many of which share exons, some reads cannot be assigned unequivocally to a specific isoform. Variability in RNA-seq data can arise, for example, from transcript length bias, library size bias (the total number of sequenced reads in each sample), and sequencing GC-content and random hexamer priming biases [7–10]. Our investigation into variability within cancer RNA-seq data reveals that bias patterns exist but cannot yet be fully explained by known sources. Moreover, different gene transcripts may exhibit different bias patterns with varying levels of complexity [11].

Currently, differential analysis of RNA-seq data mainly focuses on between-sample variability, which models the variability among biological samples in the same group [12]. Several statistical methods, such as DESeq [13], edgeR [14], EBSeq [15] and DSS [16] perform differential analysis of RNA-seq data at the gene level using statistical models, such as negative binomial distribution, to account for the variability among samples in a phenotype group. These read-count-based methods aim to improve the overall fitting of count data and the robustness against outliers. Cuffdiff 2 [6] is one of the most popular tools for differential analysis of RNA-seq data at the isoform (or transcript) level. BAM files (the binary version of sequence aligned data) are used as input and a beta negative binomial distribution accounts for the between-sample variability and read-mapping ambiguity. Cuffdiff 2 first estimates isoform expression and then detects differentially expressed isoforms based on a statistical test. It is overly conservative for detecting differentially expressed isoforms, many of which it misses [17]. Ballgown, which works together with Cufflinks [10], improves detection by flexibly selecting several statistical models [17].

Although the above-mentioned approaches have proved useful, they fail to adequately model the within-sample variability (i.e., the variability along genomic loci) of differentially expressed isoforms, which should also be taken into consideration, as we previously demonstrated [11]. Indeed, the within-sample variability is more critical at the isoform level than the between-sample variability. Gene expression consists of multiple isoforms, which thereby increases bias complexity at different locations along the gene. Cuffdiff 2 addresses within-sample variability by incorporating a fragment bias model [18] that estimates isoform expression positional and sequence-specific biases. However, the parameters of this model are estimated globally by assuming that transcripts of similar lengths have the same positional bias; this assumption is insufficient to account for the complex nature of observed within-sample variability. Moreover, after estimating isoform expression, Cuffdiff 2 applies a statistic test to detect differentially expressed isoforms, many of which may not reach statistical significance due to the large number of transcripts under consideration. Therefore, joint modeling of the variability of RNA-seq data and of the differential states of isoforms is needed for differential isoform identification.

Here, we describe and apply BayesIso, a Bayesian approach to differential analysis of RNA-seq data at the isoform level. BayesIso is based on a joint model of both the variability of RNA-seq data and differential states of isoforms. Specifically, BayesIso uses a Poisson-lognormal distribution [19] to model within-sample variability and distinct Gamma distributions [20] to model the differential expression of isoforms while accounting for the between-sample variability. Importantly, this models the within-sample variability as isoform specific and the dispersion of read counts for exons using isoform-specific parameters. Model parameters and differential states of isoforms are estimated jointly using a Markov Chain Monte Carlo (MCMC) procedure [21,22]. Simulation studies demonstrate that BayesIso significantly improves the identification of differentially expressed isoforms, especially on isoforms with moderately differential abundance.

When applied to breast cancer RNA-seq data, BayesIso identifies differentially expressed isoforms enriched in cell death, cell survival, and signaling pathways, and associated with breast cancer recurrence. Examination of differential isoforms uniquely identified by BayesIso reveals PI3K/AKT/mTOR signaling and PTEN signaling pathways responsible, at least in part, for the development of breast cancer recurrence, and a large protein-protein interaction network associated with Jak-STAT and Wnt signaling. These findings, along with further analyses of expression data, for proteins such as PIK3R2, AKT2, HSP90 and NFATC1, point to a role for isoforms in driving breast cancer recurrence.

## 2. Results

### 2.1 The BayesIso approach

**Fig 1:**
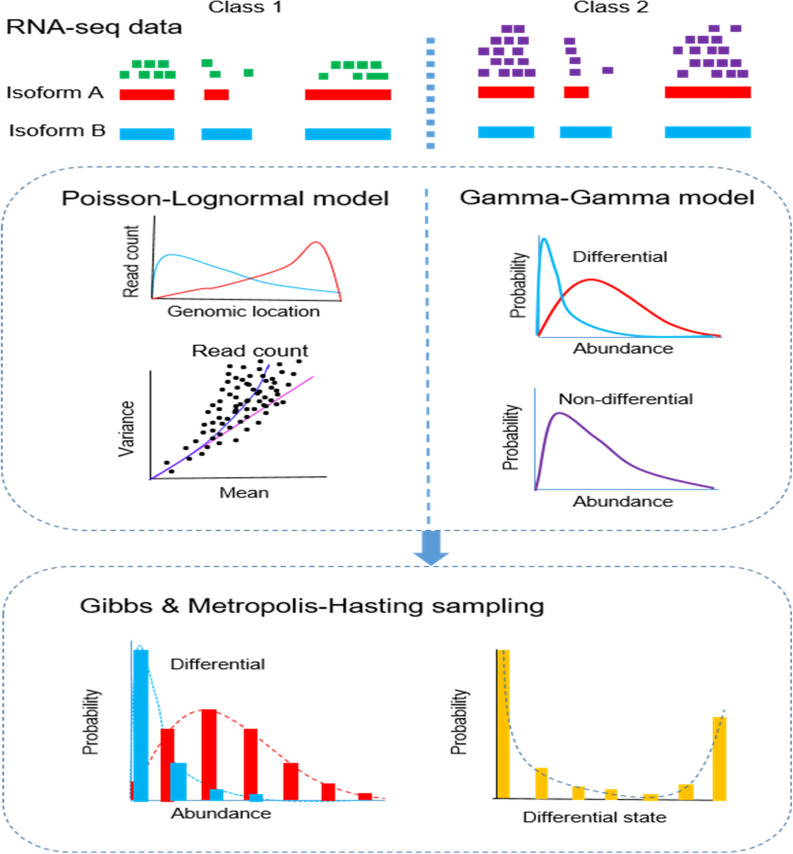
Framework of the BayesIso approach. BayesIso features a joint probabilistic model to take into account the variability in RNA-seq data and differential states of isoforms simultaneously. Specifically, a Poisson-Lognormal model is used to account for within-sample variability; a Gamma-Gamma model is used to model the isoform abundance of multiple samples (accounting for between-sample variability), embedded the differential states of isoforms as hidden variables. Finally, a Markov Chain Monte Carlo (MCMC) sampling algorithm is developed to estimate all of the model parameters and the posterior probability of the differential states.

An overview of the BayesIso approach is shown in Fig 1. As observed from real data, there are two types of variability in the RNA-seq data. The first type is ‘within-sample variability’: for an RNA-seq sample, there is a high variance of read counts arising mostly from sequencing bias along the genome. The second type is ‘between-sample variability’: among biological replicates or samples, the variance of read counts on a genomic locus is much higher than expected (i.e., the mean). A joint Poisson-Lognormal distribution models within-sample variability by representing isoform position-specific biases along the genome for each locus. A Gamma-Gamma model is used to model the differential isoform abundances of multiple samples between two classes. Specifically, differential states of the isoforms are introduced in this Gamma-Gamma model as hidden variables that control the differential isoform abundances of the samples between two classes. The joint model, in which the Poisson-Lognormal model and the Gamma-Gamma model work together, can account for the between-sample variability in addition to the within-sample variability.

Based on the joint model, a Bayesian approach is used to estimate the posterior probability of the differential state of isoforms (the hidden variable). Since the joint model is defined by a set of parameters, a Markov Chain Monte Carlo (MCMC) sampling algorithm is used to estimate the parameters and the posterior probability of the hidden variable. The MCMC sampling process consists of Gibbs sampling [23] and Metropolis-Hasting sampling [24], generating samples from the conditional distributions. By virtue of the sampling process, the (marginal) posterior distributions of the parameters and the hidden variable can be estimated (or approximated) by the samples drawn from the MCMC sampling procedure. More details about the BayesIso approach can be found in the Methods section.

### 2.2 Identifying isoforms associated with breast cancer recurrence

We applied BayesIso to breast cancer data acquired by The Cancer Genome Atlas (TCGA) project [25]. The study was designed to identify the differentially expressed isoforms associated with breast cancer recurrence. 93 estrogen receptor positive (ER+) tumors from patients were collected for this study. 61 patients were still alive with follow up longer than 5 years and labeled as ‘Alive’. 32 patients were dead within 5 years and labeled as ‘Dead’. The histogram of the survival time is shown in S1 Fig. The ‘Dead’ and ‘Alive’ groups represent the ‘early recurrence’ group and the ‘late/non recurrence’ group, respectively.

We downloaded the sequencing data (Level 1) profiled by Illumina HiSeq 2000 RNA Sequencing Version 2 from the TCGA data portal, and then performed alignment using ‘TopHat 2 (TopHat v2.0.12)’ with UCSC hg19 as the reference sequence. With the isoform structure annotation file (RefSeq genes) downloaded from the UCSC genome browser database [26], we applied our method to identify differentially expressed isoforms by analyzing samples from the ‘Dead’ group vs. the ‘Alive’ group. As observed, our model captures various bias patterns along the genomic location (S2 Fig). While the overall bias pattern of all isoforms is high in the middle, different isoform subgroups are of varying bias patterns. With a threshold set to Probability >0.75, 2,299 isoforms of 1,905 genes are identified as being differentially expressed. The histogram of the estimated probability that the isoforms are differentially expressed is shown in S3 Fig. We also calculate the SNR of the identified differentially expressed isoforms. S4 Fig shows that the SNR has a mode value around −5dB, indicating that most of the identified isoforms are moderately differentially expressed. The low mean SNR value is consistent with the high variability of expression level observed across the samples. Thus, the detection power on moderately differential isoforms is critical for differential analysis of breast cancer RNA-seq data.

### 2.3 Key pathways associated with breast cancer recurrence

**Fig 2:**
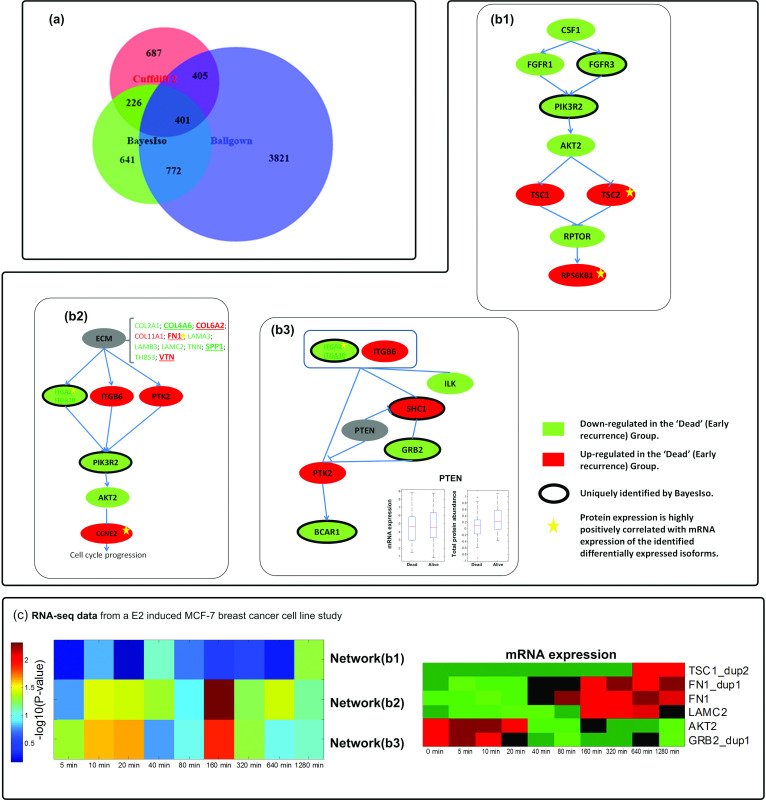
Key pathways associated with breast cancer that are uniquely identified by BayesIso. (a) Venn diagram of identified differential genes (genes with differentially expressed isoforms) by the three methods: BayesIso, Cuffdiff 2, and Ballgown. (b) Three networks of differential genes detected by BayesIso: b1 – a network related to PI3K/AKT/mTOR signaling pathway; b2 – a network related to cell cycle progression of PI3K/AKT signaling pathway; b3 – a part of PTEN signaling pathway. The color of nodes represents the expression change between the two phenotypes: green means down-regulated in the ‘Dead’ group; red mean up-regulated in the ‘Dead’ group. Genes marked by bold circle or underlined are uniquely detected by BayesIso. Genes marked by yellow star have consistent protein/phosphoprotein expression. (c) Enrichment analysis of three networks using a time-course E2 induced MCF-7 breast cancer cell line data (collected at 10 time points: 0, 5, 10, 20, 40, 80, 160, 320, 640, 1280 minutes, with one sample at each time point): left – enrichment analysis of the three networks; right – expression of transcripts with significant pattern change.

We compared BayesIso with Cuffdiff 2 and Ballgown in terms of identified differential genes. Differential genes are defined as genes with at least one differentially expressed isoform. With the criterion of *p* < 0.05 for Cuffdiff 2 and Ballgown, 1,719 and 5,399 genes, respectively,are identified as differentially expressed isoforms. Fig 2(a) shows the overlap and difference of the gene sets identified by the three methods. Cuffdiff 2 detects fewer differential genes than Ballgown does. Among the differential genes identified by BayesIso, 30% are uniquely identified by our method when compared with those identified by Cuffdiff 2 and Ballgown. The unique set of differential genes reveals several signaling pathways including the PI3K/AKT/mTOR signaling and PTEN signaling pathways (Fig 2(b1)) shows the PI3K/AKT/mTOR signaling pathway, the hyperactivation of which is known to be associated with tumorigenesis in ER positive breast cancer [27,28]. Moreover, PI3K and AKT are among the most commonly mutated genes in this breast cancer subtype; PIK3R2, a member of the PI3K protein family participating in the regulatory subunit, is detected by BayesIso as down-regulated in the ‘Dead’ group. The loss of expression of PIK3R2 is crucial to the hyperactivation of the PI3K/AKT/mTOR signaling pathway by regulating AKT2. AKT2 dysfunction inhibits the expression of TSC1 and TSC2 activates mTOR signaling, as indicated by the overexpression of RPS6KB1, a downstream target of mTOR. Overexpression of TSC2 and RPS6KB1 is further validated by their protein/phosphoprotein expression measured by reverse phase protein array (RPPA) on a subset of the TCGA breast cancer samples comprising 45 samples in the ‘Alive’ group and 27 samples in the ‘Dead’ group. Specifically, expression of NM_001114382, a differentially expressed isoform of TSC2, is positively correlated with its phosphoprotein expression at pT1462 (*p* = 0.02). Expression of NM_001272044, a differentially expressed isoform of RPS6KB1, is positively correlated with its phosphoprotein expression at pT389 (*p* = 0.0081). Note that the FPKM (expression) of NM_001272044 estimated by Cuffdiff 2 is not correlated with its phosphoprotein expression, indicating that the isoform expression estimated by BayesIso is more consistent with protein expression than Cuffdiff 2. The network shown in Fig 2(b2) reveals part of the PI3K/AKT signaling pathway leading to cell cycle progression. FN1 and ITGA2 are uniquely detected by BayesIso, which correlates with the overexpression of CCNE2 in the “Dead” group. Total protein expression values for FN1, ITGA2, and CCNE2 are highly correlated with the estimated expression of their respective isoforms. Fig 2(b3) shows part of the PTEN signaling, the underexpression of which results in hyperactivation of PI3K/AKT signaling in breast cancer [29,30]. While the mRNA expression of PTEN is not differential, total protein has a much lower expression level in the ‘Dead’ group as shown in the boxplots. BayesIso also detects SHC1, GRB2, and BCAR1, three critical components in PTEN signaling.

We validated the identified transcripts in the three networks (Fig 2(b)) using data from a time-course of estrogen (E2) induced transcription in MCF-7 breast cancer cells (RNA-seq data; GSE62789). Specifically, for each time point, we obtain the fold change of transcript expression in log2 scale compared with the sample at time 0, and used the mean of fold change as the test statistic for each network. We calculate the *p*-value for each network from a significance test where the null distributions were generated by calculating test statistics from randomly sampled gene sets of the same size of the network (100,000 iterations). Enrichment scores, defined as the negative of logarithm of *p*-value to base 10, are shown on the left panel of Fig 2(c). Two networks (Fig 2(b2) and (b3)) are enriched at early time points (<160 minutes). Moreover, the differential isoforms of TSC1, FN1, LAMC2, AKT2, and GRB2 exhibit significant expression pattern changes over time (Fig 2(c), right panel).

Note that the other genes identified by the three methods may also be associated with breast cancer. However, our initial analysis, as reported in the supplementary material (S1 Text (Section S1); S1 and S2 Tables), leads us to believe the pathways revealed by the large number of genes are diverse, hence hard to pin down their mechanistic involvement in the development of breast cancer recurrence.

### 2.4 PPI networks associated with breast cancer recurrence

We further mapped the differentially expressed genes to the Protein-Protein interaction (PPI) network from the Human Protein Reference Database (HPRD) [31], and then filtered out extremely low abundant isoforms according to the abundance relative to all of the isoforms of the same gene. With the criterion of median relative abundance >10%, 359 isoforms from 308 genes are identified as differentially expressed, among which 195 genes consist of multiple isoforms according to the annotation file with isoform structure. Furthermore, when compared with a gene-level analysis with the same criterion (‘Prob(d) > 0.75’) used to identify differentially expressed genes, 133 multiple-isoform genes are identified as differential at the isoform level but non-differential at the gene level.

Functional enrichment analysis of the 308 differentially expressed genes using Ingenuity Pathway Analysis (IPA; http://www.qiagen.com/ingenuity) reveals that many of the genes are associated with the cellular functions of proliferation, cell death, and migration. Functional enrichment analyses of the differentially expressed genes using DAVID (the Database for Annotation, Visualization and Integrated Discovery, http://david.abcc.ncifcrf.gov/home.jsp) shows that the identified gene set is enriched in several KEGG signaling pathways and functional clusters listed in Table 1.

**Table 1:** Enriched KEGG pathways and functional clusters.

Term	Count	*P* Value	Genes
Pathways in cancer	27	6.84E−06	TRAF1, HSP90AB1, FGFR3, ERBB2, MLH1, MMP1, TGFB2, FLT3LG, FOS, LAMB3, CDKN2A, PIK3R2, AKT2, RET, HSP90AA1, IL8, FLT3, CREBBP, BRCA2, HGF, MAPK10, MECOM, BIRC3, COL4A6, LAMA3, ETS1, LAMC2
Insulin signaling pathway	15	6.45E−05	IRS2, PTPRF, SOCS3, RPS6KB1, IGF2, MAPK10, PPP1CA, PRKAR2A, PDPK1, TSC1, TSC2, SH2B2, PRKACB, AKT2, PIK3R2
NOD-like receptor signaling pathway	10	1.06E−04	HSP90AB1, CARD8, HSP90AA1, IL8, PSTPIP1, CCL8, NAIP, MAPK10, CASP1, BIRC3
Focal adhesion	18	1.41E−04	CAV2, ERBB2, VTN, COL2A1, HGF, MAPK10, BIRC3, COL4A6, PPP1CA, LAMB3, PDPK1, LAMA3, CCND3, ITGB6, LAMC2, MYLK, AKT2, PIK3R2
Cytokine-cytokine receptor interaction	21	1.49E−04	IL2RB, IL8, IL6ST, FLT3, CCR1, TNFRSF17, CCL8, CCL19, CNTFR, CXCR3, HGF, TNFRSF4, TGFB2, FLT3LG, LEP, OSM, CXCL13, CCL21, TNFRSF18, NGFR, BMPR1B
Progesterone-mediated oocyte maturation	11	2.92E−04	HSP90AB1, ADCY2, HSP90AA1, ADCY6, IGF2, MAPK10, PRKACB, CDC27, CDC25B, PIK3R2, AKT2
Jak-STAT signaling pathway	13	0.0029	IL2RB, SOCS3, IL6ST, CREBBP, PIM1, SOCS7, CNTFR, OSM, LEP, CCND3, STAM, AKT2, PIK3R2
mTOR signaling pathway	7	0.0051	PDPK1, TSC1, TSC2, IGF2, RPS6KB1, PIK3R2, AKT2
Chemokine signaling pathway	12	0.0302	ADCY2, IL8, CCL21, CXCL13, CCR1, ADCY6, CCL8, CCL19, CXCR3, PRKACB, PIK3R2, AKT2
MAPK signaling pathway	14	0.0704	FGFR3, TAOK1, RELB, DUSP10, NR4A1, MAPK10, MECOM, TGFB2, CDC25B, FOS, DUSP2, MAPK8IP3, PRKACB, AKT2
Wnt signaling pathway	9	0.0967	PPP2R1B, CCND3, BTRC, CREBBP, MMP7, MAPK10, PRKACB, FOSL1, NFATC1
Response to hormone stimulus	30	5.45E−10	CAV2, ADCY2, ERBB2, ADCY6, RPS6KB1, TGFB2, FOS, PRKAR2A, PDPK1, SH2B2, PRKACB, FOSL1, ADAM9, AKT2, IRS2, EGR2, ACTA1, CRYAB, SOCS3, MAP1B, ESR1, SOCS7, BRCA2, IGF2, MMP13, BRCA1, LEP, GRB10, TSC1, VLDLR
Regulation of apoptosis	45	2.45E−09	TRAF1, FASTK, ERBB2, CLU, MLH1, COL2A1, TLR4, ADORA1, TNFRSF4, TGFB2, CDKN2A, BAG1, ALB, AGT, TNFRSF18, CASP1, CD5, TOP2A, FOSL1, ADAM9, IL2RB, CARD8, SGK3, PTPRF, CD3E, CRYAB, SOCS3, PIM1, ESR1, BIRC6, BRCA2, NR4A1, IGF2, MBD4, HGF, BIRC3, BRCA1, TAX1BP1, SOD2, CASP14, ETS1, LCK, NAIP, NGFR, FAIM2
Immune system development	20	4.57E−06	CD3D, FLT3, CD3E, RELB, TIRAP, MLH1, BRCA2, SOD2, TGFB2, FLT3LG, CXCL13, LCK, ZAP70, HOXA9, CD4, PBX1, CD79A, ACIN1, BLNK, MED1
Cytoskeletal protein binding	26	5.58E−05	WASF2, FHL3, TPM2, PACSIN1, SYN1, PIP, PSTPIP1, CDK5RAP2, PLEC, RET, ACTA1, EPB41, CRYAB, MAP1A, MAP1B, BRCA2, TNNI3, BRCA1, S100B, NEB, LASP1, SDCBP, MAPK8IP3, SYNM, DST, MYLK
Regulation of cell adhesion	12	1.25E−04	TESC, CDKN2A, CD36, LAMA3, TSC1, IL8, ERBB2, TSC2, VTN, DPP4, ADAM9, TGFB2

**Fig 3:**
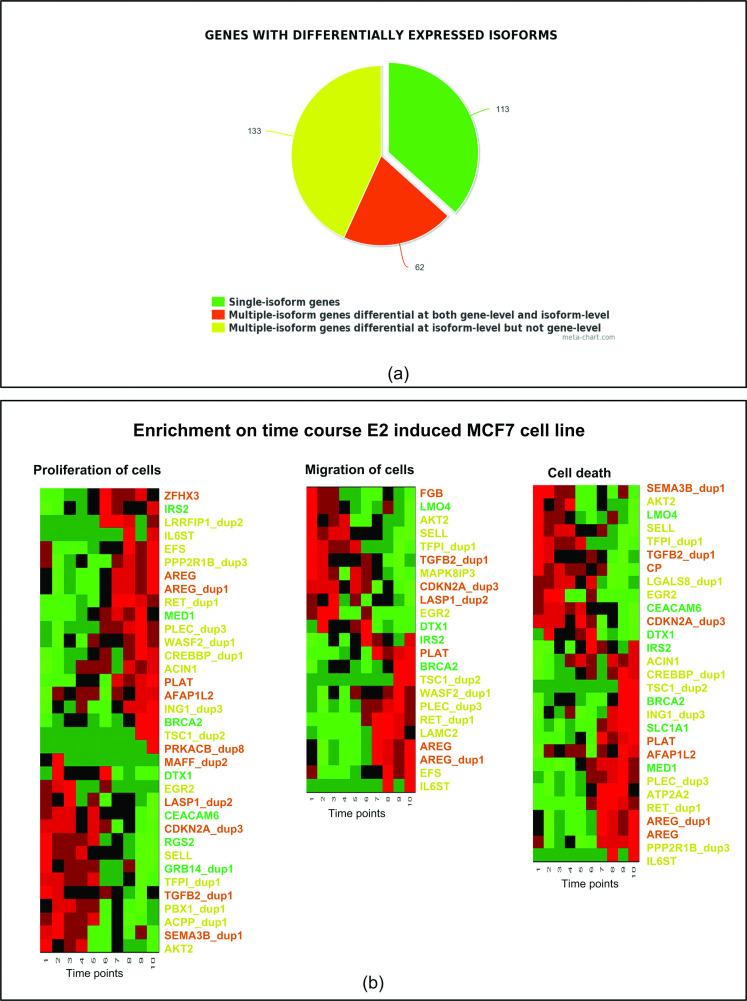
Enrichment analysis of the identified differentially expressed isoforms overlapped with PPI network. **(a)** The identified genes are categorized as single-isoform genes (genes with only one isoform) and multiple-isoform genes (genes with multiple isoforms). The multiple-isoform genes are further divided into two groups: differential at both gene-level and isoform-level, differential at the isoform level only. **(b)** Heatmaps of genes associated with proliferation of cells, migration of cells, and cell death, showing expression pattern change in a time-course E2 induced MCF-7 cell line data. The gene symbols of the heatmaps are color-coded according to the grouping in **(a)**.

We also validated the associated sets of isoforms on the estrogen (E2) induced time-course dataset (RNA-seq data; GSE62789). Both sets of isoforms associated with cell proliferation and migration are significantly enriched (*p* = 0.043 and *p* = 0.021, respectively); the *p*-value of the isoforms associated with cell death is borderline (*p*= 0.07). As shown in Fig 3, the expression of several isoforms, such as AKT2 and TSC1, changes significantly across time, implicating these genes and their isoforms in breast cancer development and recurrence. Details of the validation study can be found in S1 Text, Section S2.

**Fig 4:**
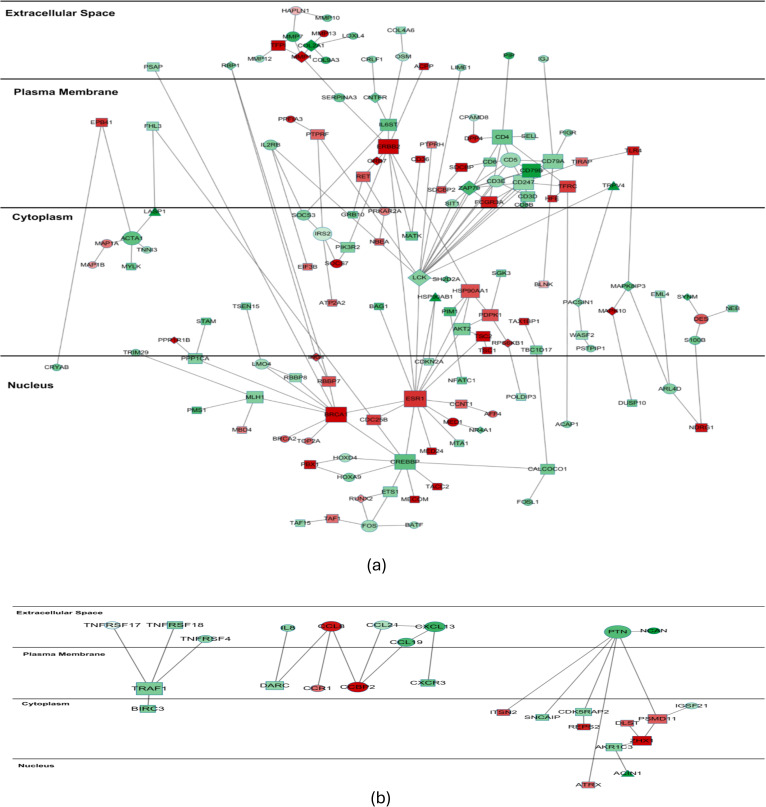
PPI networks of the identified differentially expressed genes. *Node color* denotes the fold change: genes overexpressed in the ‘Dead’ group are shown in red; genes overexpressed in the ‘Alive’ group are shown in green. *Node shape* denotes the isoform information of the gene: round nodes are genes with single isoforms; rectangle nodes are genes with multiple isoforms but only one isoform is differentially expressed; Diamond nodes are genes with multiple differentially expressed isoforms which are all up- or down-regulated; Triangle nodes are gene with multiple differentially expressed isoforms which are regulated in the opposite direction. *Node size* denotes the node degree.

The protein-protein interaction (PPI) networks of the differentially expressed genes are shown in Fig 4, where Fig 4(a) is the major connected network and Fig 4(b) represents small, isolated networks. In the PPI network of 308 genes, several hub genes (ESR1, BRCA1, CREBBP, ERBB2, and LCK) are known to play critical roles in breast cancer development. Also important are TNFRSF17, TNFRSF18, TNFRSF4, members of the Tumor Necrosis Factor Receptor superfamily that bind to various TRAF family members and can regulate tumor cell proliferation and death [31]. Moreover, from the functional enrichment analysis using DAVID, the genes participate in several signaling pathways including Jak-STAT, mTOR, MAPK, and Wnt signaling. Studies on the Jak-STAT and mTOR signaling pathways have established their roles in key processes that contribute to malignancy such as proliferation, apoptosis, and migration [32,33].

**Fig 5:**
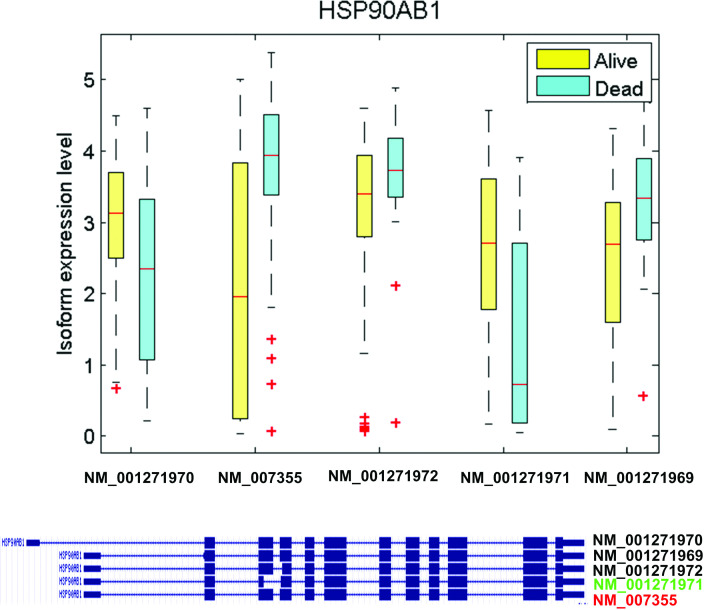
Estimated abundance of isoforms of HSP90AB1. The box plot shows the estimated expression level of the isoforms in the samples of the two phenotypes: ‘Alive’ and ‘Dead’. Isoform 2 is detected as overexpressed in the ‘Dead’ group (‘early recurrence’); isoform 4 is detected as overexpressed in the ‘Alive’ group (‘late recurrence’).

Many genes associated with the signaling pathways are differential at the isoform level but not at the gene-level. Genes that reflect isoform-only level differential expression include PDPK1, TSC1, TSC2, PIK3R2, and AKT2 (mTOR signaling), and HSP90AA1 and HSP90AB1 (PI3K/AKT signaling). Thus, accurate and robust isoform-level differential analysis is essential and provides critical information when studying biological mechanisms associated with cancer recurrence. While HSP90AA1 has two isoforms from alternative splicing, only NM_005348 (RefSeq_id) is overexpressed in the ‘Dead’ group. HSP90AB1 has five isoforms, among which NM_007355 is detected as overexpressed in the ‘Dead’ group, whereas NM_001271971 is overexpressed in the ‘Alive’ group (Fig 5). HSP90AA1 and HSP90AB1 are Heat Shock Proteins (HSPs) that play an important role in tumorigenesis [34,35]. Overexpression of HSP90AA1 and HSP90AB1 can affect cancer cell viability and provide an escape mechanism from treatment-induced apoptosis. Functional analysis using IPA implicates the down-regulation of HSP90AB1 in activation of cell death within immune cells [36]. Collectively, these findings strongly implicate the changes in different isoform expression patterns in several key functions that directly affect cancer development.

### 2.5 Upregulated/downregulated pathways associated with early/late recurrence

We further divided the identified genes into two groups according to their expression pattern. 125 genes are overexpressed in the ‘Dead’ group, 172 genes are overexpressed in the ‘Alive’ group, and the remaining 11 genes have multiple isoforms with inconsistent expression patterns. Genes overexpressed in the ‘Dead’ group are labeled as ‘up-regulated’ while those overexpressed in the ‘Alive’ group are labeled as ‘down-regulated’. We performed a functional enrichment analysis on each group using IPA for identifying the enriched pathways of the up-regulated and down-regulated gene sets, respectively (Table 2). Pathways associated with cellular metabolism, survival and cell cycle are up-regulated, while immune response genes are down-regulated in tumors that recur early.

**Table 2:** Enriched pathways on up-regulated and down-regulated genes/isoforms.

Up-regulated in early recurrence		Down-regulated in early recurrence	
Ingenuity Canonical Pathways	Molecules	Ingenuity Canonical Pathways	Molecules
PPARα/RXRα Activation	CD36, ADCY6, MED1, LPL, MED24, HSP90AB1, PRKACB, HSP90AA1, PRKAR2A	**Glucocorticoid Receptor Signaling**	TAF15, CD3E, SLPI, BAG1, TGFB2, VIPR1, PRKACB, NFATC1, AKT2, CXCL8, FOS, AGT, PIK3R2, CD3D, CREBBP, HSP90AB1,CD247
AMPK Signaling	PDPK1, TSC2, LEP, PPP2R1B, PRKACB, TSC1, RPS6KB1, PRKAR2A	**T Cell Receptor Signaling**	FOS, CD3E, CD8B, PIK3R2, LCK, CD3D, BMX, CD4, CD247, NFATC1, ZAP70
LXR/RXR Activation	TLR4, CD36, VTN, CLU, LPL, AMBP, ALB	**Role of NFAT in Regulation of the Immune Response**	CD3E, CD79B, NFATC1, AKT2, FOS, CABIN1, PIK3R2, CD3D, LCK, CD4, CD247, CD79A, ZAP70
PI3K/AKT Signaling	PDPK1, TSC2, HSP90AB1, PPP2R1B, TSC1, HSP90AA1, RPS6KB1	**iCOS-iCOSL Signaling in T Helper Cells**	CD3E, PIK3R2, LCK, CD3D, IL2RB, CD4, CD247, NFATC1, AKT2, ZAP70
Insulin Receptor Signaling	PDPK1, TSC2, PTPRF, PRKACB, TSC1, RPS6KB1, PRKAR2A	**PKC**θ **Signaling in T Lymphocytes**	FOS, CD3E, PIK3R2, LCK, CD3D, CD4, CD247, NFATC1, ZAP70
Cell Cycle: G2/M DNA Damage Checkpoint Regulation	TOP2A, BRCA1, BTRC, CDC25B	**NF-κB Signaling**	FGFR3, TNFRSF17, NGFR, RELB, PIK3R2, LCK, CREBBP, PRKACB, AKT2, ZAP70

CD36 is a multi-ligand cell surface transmembrane receptor that regulates apoptosis, adipocyte differentiation, cellular metabolism, immunity and angiogenesis [37]. CD36 expression, which can be regulated by estrogen and anti-estrogens [38], has been associated with mammary density and clinical outcome [39]. HSP90AA1 (HSP90) is a chaperone protein and its inhibition impaired the emergence of resistance to hormone antagonists both in cell culture and in mice [40]. Antiestrogen resistance is sustained by up-regulation of autophagy, a cellular cannibalistic process, that is closely regulated by mTOR [41,42]. Upstream of mTOR, AMPK signaling controls growth factors and energy signaling cascades including PI3K/AKT and insulin signaling [43–45], and also regulates autophagy [41,42].

TOP2A (topoisomerase II alpha) is an enzyme that catalyzes the topological DNA changes needed during the multistep process of cell division [46]. In breast cancer, TOP2A expression correlates significantly with ER, Ki-67, and HER2 expression [47]. Aberrations of chromosome 17q12-q22 have been reported in breast cancer and this locus incorporates the TOP2A gene along with HER2 [48]. Several of the cytotoxic drugs routinely used in the management of advanced breast cancer target topoisomerases including doxorubicin and epirubicin [49]. Overexpression of topoisomerase may reduce the efficacy of these anthracyclines, leading to drug resistance and early recurrence in some patients [50]. Expression of NFATC1, a member of a family of transcription factors that regulate the immune system, is down-regulated in early-recurrent breast tumors [51]. This down-regulation may partly explain the decrease in T cell helper-mediated antitumor activity seen in some breast cancers [52].

## 3. Discussion

RNA-seq data make it possible for large-scale isoform-level differential analysis yet also post remarkable challenges due to high variability and uncertainty of read assignment at the transcript level. We have developed a Bayesian approach, BayesIso, for the identification of differentially expressed isoforms. A hierarchical model, with differential states as hidden variables, is devised to account for both between-sample variability and within-sample variability. Specifically, a Poisson-Lognormal model is used to model the within-sample variability specific to each transcript. The expression level of transcripts is modeled to follow a Gamma distribution so as to capture the between-sample variability, including both over-dispersion and under-dispersion, by the model parameters. The shape parameter of the Gamma distribution is further assumed to follow a second Gamma distribution. Differential states of the transcripts are embedded into the Gamma-Gamma model as hidden variables, affecting the distribution of transcript expressions in each group or condition.

We have applied BayesIso to breast cancer RNA-seq data to identify differentially expressed isoforms associated with breast cancer recurrence. The diverse bias patterns along transcripts and the generally low differential level have been observed from the real breast cancer data, indicating their importance in differential analysis of RNA-seq data. The differentially expressed isoforms detected by BayesIso are enriched in cell proliferation, apoptosis, and migration, uncovering the mechanism related to breast cancer recurrence. Moreover, the unique set of differential genes identified by BayesIso has helped reveal several signaling pathways such as the PI3K/AKT/mTOR signaling and PTEN signaling pathways. The identified down-regulated genes in the early recurrence group, e.g., NFATC1, participate in the immune system, which may indicate the role of immune system in breast cancer recurrence.

As a final note, it is a non-trivial task to model the sequencing bias for RNA-seq data analysis. The bias patterns are complicated and cannot be well explained by known sources. In the BayesIso method, we have used a flexible model to account for the bias independent of any particular pattern. However, we have also observed that certain bias patterns (such as bias to the 3′ end, or high in the middle) occur more frequently than others. Moreover, we have further observed that the bias patterns may be affected by the expression level. In the future work, we will incorporate certain bias patterns as prior knowledge into the model, which can help estimate the bias pattern of some isoforms more accurately hence improve the performance in differential analysis of isoforms.

## 4. Methods

### 4.1 Model description of the BayesIso approach

Let $y_{g,t,i,j}$ represent the observed counts that fall into the *i*^th^ (1 ≤ *i* ≤ *I*_*g*_) exon region of isoform *t* (1 ≤ *t* ≤ *T*) of gene *g* (1 ≤ *g* ≤ *G*) in sample *j* (1 ≤ *j* ≤ *J*). *T* is the number of isoforms of gene *g* given by the annotation information. *I*_*g*_ is the number of exons in gene *g*. *G* is the total number of genes. *J* = *J*_1_ + *J*_2_ is the total number of samples, where *J*_1_ and *J*_2_ denote the number of samples in phenotype 1 and 2, respectively. Since one gene may have multiple isoforms, *y*_*g,i,j*_, the observed counts in the exon region, is the combination of all potential isoforms, as defined in Eq. (1):


$$y_{g,i,j}=\sum_{t}s_{g,t,i}y_{g,t,i,j},$$ (1)

where $s_{g,t,i}$ is a binary value indicating whether exon *i* is included in isoform *t* of gene *g*. At the isoform level, we use a Poisson-Lognormal regression model to account for the within-sample variability of RNA-seq data. $y_{g,t,i,j}$ follows a Poisson distribution with mean $\gamma_{g,t,i,j}$:


$$y_{g,i,j}\sim \text{Poiss}\left(\gamma_{g,t,i,j}\right).$$ (2)

According to the Poisson-Lognormal model [19],


$$\gamma_{g,t,i,j}=\;x_{g,i,j}\beta_{g,t,j}\text{exp}\left(U_{g,t,i}\right),$$ (3)

$$U_{g,t,i}\sim \;N\left(0,\tau_{g,t}\right),\;\text{s}.\text{t}.\sum_{i}U_{g,t,i}=0\;,$$ (4)

$$\tau_{g,t}\sim \;Gamma\left(a,b\right),$$ (5)

where $\beta_{g,t,j}$ is the true expression level of isoform *t* of gene *g* in sample *j*. $x_{g,i,j}$ is the length of the *i*^th^ exon weighted by the library size of sample *j*. *U*_*g,t,i*_ is a model parameter representing the within-sample variability (or dispersion) for exon *i* of isoform *t* of gene *g*. Thus, the dispersion of different loci, exons of the isoforms, is modeled by different parameters. Precision parameter $\tau_{g,t}\;$ controls the overall degree of within-sample variability.

We use a Gamma-Gamma model [53] to model the expression level *β*_*g,t,j*_ across samples collected from two phenotypes. The differential state, as a hidden variable in this Bayesian model, affects the distribution of *β*_*g,t,j*_ among samples in each of the two phenotypes. $d_{g,t}$, a binary value, indicates the differential state of isoform  t of gene g, where $d_{g,t}=1$ means isoform *t* of gene g is differentially expressed; $d_{g,t}=0$, otherwise. Note that the between-sample variability is captured by the Gamma distribution. From the Gamma-Gamma model, the isoform expression level *β*_*g,t,j*_ is given by:


$$\beta_{g,t,j}\sim Gamma\left(\alpha ,\lambda_{g,t}\right),\text{ if }d_{g,t}=0$$ (6)

$$\lambda_{g,t}\sim Gamma\left(\alpha_{0},v\right);$$ (7)

$$\text{or, }\beta_{g,t,j_{1}}\sim Gamma\left(\alpha ,\lambda_{g,t}^{\left(1\right)}\right),\;\beta_{g,t,j_{2}}\sim Gamma\left(\alpha ,\lambda_{g,t}^{\left(2\right)}\right),\text{ if }d_{g,t}=1$$ (8)

$$\lambda_{g,t}^{\left(1\right)},\lambda_{g,t}^{\left(2\right)}\sim Gamma\left(\alpha_{0},v\right)$$ (9)

$$\text{and, }v\sim Gamma\left(a_{0},b_{0}\right)$$ (10)

where *α* is the shape parameter; $\lambda_{g,t}$ is the rate parameter that depends on differential state $d_{g,t}$. If $d_{g,t}=0$, $\lambda_{g,t}^{\left(1\right)}=\lambda_{g,t}^{\left(2\right)}=\lambda_{g,t}$; if $d_{g,t}=1$, $\lambda_{g,t}^{\left(1\right)}\neq \lambda_{g,t}^{\left(2\right)}$. $\lambda_{g,t}$ is further assumed to follow a Gamma distribution with shape parameter α_0_ and rate parameter *ν*. In marked contrast to existing methods like Cuffdiff 2 that uses statistical tests to identify differentially expressed isoforms, the differential states of isoforms are introduced and modeled in the proposed joint model of BayesIso. A joint estimation of the differential states with other model parameters is accomplished by a Markov Chain Monte Carlo (MCMC) sampling method as described in detail in the next section.

### 4.2 The MCMC algorithm used in BayesIso

Due to the complexity of the joint model, it is challenging to estimate directly the model parameters and the hidden variables (i.e., the differential states, **d** = [$d_{g,t}$]). We have designed a Markov Chain Monte Carlo (MCMC) method to estimate the parameters and the hidden variables (**d**). The MCMC sampling process is a combination of Gibbs sampling and Metropolis-Hasting (M-H) sampling, with which as many samples as possible can be generated or drawn from the conditional distributions. By virtue of the sampling process, the marginal posterior distributions of the parameters and the hidden variable can be approximated by the samples drawn from the MCMC sampling procedure. Next, we will describe the MCMC algorithm and the associated conditional distributions.

Based on the assumption that the expression levels of the transcripts are independent, the likelihood of the observation $y_{g,i,j}$ given all the parameters is $P(y_{g,i,j}|\beta_{g,j},U_{g,t,i})\sim \text{Poiss}\left(\underset{t}{\sum }\left(s_{g,t,i}\gamma_{g,t,i,j}\right)\right)$. Thus, the conditional (posterior) distributions of the parameters $U_{g,t,i}$ and $\tau_{g,t}$ (of the Poisson-Lognormal model) can be derived as follows:

$$\begin{array}{l} P\left(U_{g,t,i}\text{|}y_{g,i},\beta_{g,j},\tau_{g,i}\right)\sim \underset{j}{\prod }P\left(y_{g,i}|U_{g,t,i},\beta_{g,j}\right)\times P\left(U_{g,t,i}|\tau_{g,t}\right)\\ \sim \underset{j}{\prod }\left({\left(\underset{t}{\sum }s_{g,t,i}\beta_{g,t,j}x_{g,i}\text{exp}\left(U_{g,t,i}\right)\right)}^{y_{g,i,j}}\times \text{exp}\left(-\underset{t}{\sum }\left(s_{g,t,i}\beta_{g,t,j}x_{g,i}\text{exp}\left(U_{g,t,i}\right)\right)\right)\right),\\ \times \text{exp}\left(-\frac{\tau_{g,t}{\left(U_{g,t,i}\right)}^{2}}{2}\right) \end{array}$$ (11)

$$\begin{array}{l} P\left(\tau_{g,t\;}\text{|}U_{g,t}\right)\sim \underset{g,t,i}{\prod }P\left(U_{g,t,i}|\tau_{g,t\;}\right)\times P\left(\tau_{g,t}\right)\;\\ \sim \;Gamma\left(a+\frac{\sum_{i}s_{t,g,i}}{2},b+\underset{i}{\sum }\frac{{\left(U_{g,t,i}\right)}^{2}}{2}\right)\;. \end{array}$$ (12)

Similarly, the conditional posterior distributions of the parameters **β**, **λ**, *α*, *α*_0_, *ν* and **d** for the Gamma-Gamma model can also be derived. The details can be found in S1 Text, Section S3.

With the conditional posterior distributions derived, the MCMC algorithm is designed with the steps for Gibbs sampling and Metropolis-Hasting (M-H) sampling. Note that M-H sampling is used to sample the parameters without conjugate priors, while Gibbs sampling is used to sample the parameters with conjugate priors. The MCMC algorithm can be summarized as follows:

INPUT**:** Observed read counts *y*, library size weighted isoform structure x, number of iterations N

OUTPUT**:** Estimates of all of the parameters and the differential state **d** in the joint Bayesian model

#### Algorithm

**Step 1.** Initialization: each parameter is set an arbitrary value and non-informative prior knowledge is used for the parameters.

**Step 2.** Draw samples iteratively from the conditional distributions of parameters **β**, **U**, **τ** (in the Poisson-Lognormal model) and parameters **λ**, **α**, **α**_0_, **ν** and **d** (in the Gamma-Gamma model). Perform the following sampling steps for N iterations:

• Use Gibbs sampling to draw samples of **β**, **τ**, **λ**, **ν** from their conditional distributions that follow standard probability distributions;
• Use Metropolis-Hasting (M-H) sampling to draw samples of **U**, **d**, **α**, **α**_0_ from their conditional distributions in sequence. Since these parameters do not have conjugate priors, M-H sampling is used to approximate their posterior distributions.

**Step 3.** Estimate differential state **d** as well as other parameters **β**, **U**, **τ**, **λ**, *α*, *α*_0_, *ν* from the samples, after the burn-in period, generated from the MCMC procedure.

### 4.3 Performance evaluation of the BayesIso Approach

We conducted a comprehensive study to evaluate the performance of BayesIso, focusing on differential analysis of RNA-seq data at the isoform level. We ran our experiments using genes with an increasing number of isoforms, starting from genes with two isoforms. For each experiment, gene sets were randomly selected from the annotation file from the UCSC genome browser database (version: GRCh37/hg19; http://genome.ucsc.edu/). Multiple synthetic data sets with varying model parameters were generated using our simulator that produced aligned reads in the ‘BAM’ format. We compared BayesIso with two existing methods: Cuffdiff 2 (version 2.2.1) [6] and Ballgown (version 1.0.4) [54], for isoform-level differential analysis of RNA-seq data.

Specifically, the performance of BayesIso was evaluated based on its accuracies in abundance quantification and differential isoform identification, respectively. The performance of BayesIso was compared with Cufflinks and Cuffdiff 2; BayesIso exhibits a consistent and improved performance over competing methods in cases of different within-sample variability and bias pattern. S4 and S5 Tables summarize the results from the performance comparison study, showing the advantage of BayesIso over existing methods for differential analysis of isoforms. More details about the performance on abundance quantification and differential analysis of isoforms can be found in S1 Text, Sections S4 & S5

We also generated synthetic data using a RNA-seq simulator (RNAseqReadSimulator [25]) to test the performance of the competing methods. 1,000 isoforms from 500 genes were randomly selected for the experiment, where 498 isoforms were differentially expressed. Consistent with previous comparison, our method has achieved the highest overall performance measured by F-score (S8 Table). Cuffdiff 2 gives rise to a very high precision, yet the recall is very low. Note that BayesIso is of a higher detection power (with a much higher recall) on the moderately differential isoforms (−3dB < SNR < −1dB; see S1 Text, Section S6 for more details).

We further used real RNA-seq benchmark datasets to evaluate the performance of the competing methods on differential analysis of RNA-seq data. The datasets are part of the MicroArray Quality Control Project (MAQC) project for benchmarking and characterize RNA-seq technology [55,56]. Using RNA spike-ins and validated expression of 1,000 genes by qRT-PCR, the performance of BayesIso was benchmarked and compared with existing methods. The results further support that the joint model employed in BayesIso has resulted in an improved overall performance for differential analysis of isoforms as shown in S13 and S14 Figs; more details of the ROC study and the precision-recall study can be found in S1 Text, Section S7.

## Supporting information

S1 Text Supplementary material: methods, performance evaluation and breast cancer study. (DOCX)

S1 Fig Histogram of patients’ survival time: the ‘Dead’ group is shown in red; the ‘Alive’ group is shown in blue. (TIF)

S2 Fig Estimated bias patterns of the sequencing reads. The mean bias pattern of all the isoforms is shown by the red curve in the up-left figure. However, different sets of isoforms exhibit varying bias patterns. The three blue curves show the mean bias patterns of different groups of isoforms. The isoforms are grouped according to their bias patterns.
(TIF)

S3 Fig Histogram of estimated probability that the isoforms are differentially expressed. Red line denotes Prob(d=1) = 0.75.
(TIF)

S4 Fig Histogram of SNR of the differentially expressed isoforms. (TIF)

S5 Fig Histogram of estimated abundance of TNFSF10 in samples in the two groups. Blue bars denote the abundance in the ‘Alive’ group, and the blue curve denotes the fitting of the blue bars with a gamma distribution. Red bars denote the abundance in the ‘Dead’ group, and the red curve denotes the fitting of the red bars with a gamma distribution.
(TIF)

S6 Fig Performance comparison on abundance quantification. (a) Different overall within-sample variability; (b) different bias patterns along the genomic location. Average correlation coefficient between the estimated abundance and the true abundance of the isoforms is used to evaluate the performance.
(TIF)

S7 Fig Histogram of the SNR of all truly differentially expressed isoforms. Red line denotes SNR = −1 dB; green line denotes SNR = −3 dB.
(TIF)

S8 Fig Results of differential analysis using the three competing methods: (a) histogram of the probability for the isoforms to be differentially expressed estimated by BayesIso; (b) histogram of the *p*-value calculated by Cuffdiff 2; (c) histogram of the *p*-value calculated by Ballgown. (TIF)

S9 Fig Performance comparison on abundance quantification on different groups of isoforms. The average correlation coefficients of all of the isoforms of the three competing methods are listed by the left three bars, while the performance on the three groups of differentially expressed isoforms and the non-differential isoforms are shown by the other 4 groups of bars.
(TIF)

S10 FigSequencing bias along the genomic location.(a) Four different bias patterns presented by the curves are simulated by varying the sequencing probabilities according to genomic location. (b) Estimated biased patterns indicated by $exp\left(U\right)$ of the four groups of isoforms.(TIF)

S11 Fig Histogram of the SNR of the truly differential isoforms: red line denotes SNR = −1 dB; green line denotes SNR = −3 dB. (TIF)

S12 Fig Results of differential analysis using the three competing methods: (a) histogram of the probability for the isoforms to be differentially expressed estimated by BayesIso; (b) histogram of the *p*-value calculated by Cuffdiff 2; (c) histogram of the *p*-value calculated by Ballgown. (TIF)

S13 Fig Performance comparison on differential analysis using the SEQC dataset benchmarked by ERCC RNAs. (TIF)

S14 Fig Performance comparison on differential analysis on MAQC data with TaqMan qRT-PCR measurements as benchmark: (a) overall performance evaluated by F-score; (b) performances of recall and precision, respectively. (TIF)

S1 Table Enriched Ingenuity canonical pathways obtained from genes identified by BayesIso, Cuffdiff 2 and Ballgown. (XLSX)

S2 Table Enriched KEGG pathways obtained from genes identified by BayesIso, Cuffdiff 2 and Ballgown. (XLSX)

S3 Table Number of isoforms grouped according to SNR. (XLSX)

S4 TablePerformance comparison on differential analysis at varying parameters *α* or *α*_0_ (while other parameters are fixed).
In general, the less *α* is, the lower abundance of isoform is; the less $\alpha_{0}$ is, the more differentially expressed isoforms are. The precision, recall, and F-score are calculated in terms of mean values from 5 experiments. (Note that the number of isoforms is set as 2 for the genes studied in this experiment).(XLSX)

S5 Table Performance comparison on differential analysis at different SNR levels. (XLSX)

S6 Table Performance comparison on differentially expressed isoform detection on genes with 3, 4, and 5 isoforms, as well as genes with single isoform. (XLSX)

S7 Table Number of isoforms grouped according to SNR. (XLSX)

S8 Table Performance comparison on differential analysis at different SNR levels. (XLSX)
