# Supplementary material for: Bayesian identification of differentially expressed isoforms using a novel joint model of RNA-seq data
Source: PLoS Comput Biol. 2025 Jan 31;21(1):e1012750. doi: 10.1371/journal.pcbi.1012750 (PMC11819608; doi:10.1371/journal.pcbi.1012750)
Supplement: S1 Text — (DOCX) [file pcbi.1012750.s001.docx]

Supplementary Material

**Bayesian identification of differentially expressed isoforms using a novel joint model of RNA-seq data**

Xu Shi^1^, Xiao Wang^1^, Lu Jin^2^, Leena Hilakivi-Clarke^2^, Robert Clarke^2^, Andrew F. Neuwald^3^, Jianhua Xuan^1^,*

**Table of Contents**

[Supplementary Material 1](#_Toc187246537)

[S1. Experiment on real RNA-seq data to study breast cancer recurrence 2](#_Toc187246538)

[S2. A validation study on MCF7 breast cancer cell line 3](#_Toc187246539)

[S3. Supplemental to Methods 5](#_Toc187246540)

[S4. Performance evaluation on abundance quantification 9](#_Toc187246541)

[S5. Performance evaluation on identification of differential isoforms 11](#_Toc187246542)

[S6. Simulation study using RNAseqReadSimualtor 14](#_Toc187246543)

[S7. Performance Comparison on Real RNA-seq Data 15](#_Toc187246544)

[References 19](#_Toc187246545)

# S1. Experiment on real RNA-seq data to study breast cancer recurrence

We collected a set of estrogen receptor positive (ER+) breast cancer tumor samples from TCGA to study the mechanism underlying recurrence. The tumor samples were divided into two groups according to the survival time. Specifically, tumor samples from patients who were still alive with follow up time longer than 5 years were grouped as ‘Alive’, indicating ‘late recurrence’, while those from patients dead within 5 years were grouped as ‘Dead’, indicating ‘early recurrence’. S1 Fig shows the distribution of the ‘survival time’ of the patients.

Note that the study is focused on a subset of patients of early and late recurrence. To make sure the patients selected with early/late recurrence, we use a stringent criterion as follows: (1) early recurrent patients: they were followed up **within 5 years** and **dead** (resulting in only 32 patients); similarly, (2) late recurrent patients: they were followed up **longer than 5 years** and **alive** (resulting in 61 patients). This criterion may be too strict, but that helps us design a focused study to uncover a subset of molecular mechanisms driving breast cancer recurrence. Although TCGA collected more than 1000 samples, the vital status (alive or dead) greatly limits the number of selected samples for this focused study.

We applied our proposed method, BayesIso, to detect differentially expressed isoforms comparing the two groups of samples. S2 Fig shows the estimated bias patterns of several sets of isoforms, which demonstrates that our proposed model captures various bias patterns. The estimated probability for the isoforms to be differential expressed is shown in S3 Fig with ‘Prob(d=1) > 0.75’ as the criterion to detect differentially expressed isoforms. S4 Fig is the histogram of the SNR of the identified differentially expressed isoforms, from which we can see that the differential level of the isoforms are generally not so significant with mode around SNR = -4dB. Our proposed method, BayesIso, was effective in detecting moderately differential isoforms by virtue of estimating the differential state jointly with inferring the distribution of the abundance under the two phenotypes in a Bayesian framework. Taking TNFSF10 as an example (as shown in S5 Fig), although the SNR is quite low, its abundance in samples under the two phenotypes is more likely to be from two different gamma distributions rather than from one distribution. Thus, it is more likely to be differential.

It is worth noting that currently, the threshold is a user-defined value (probability > 0.5) for selecting a final set of differential genes/isoforms. In our study, to avoid too many genes/isoforms for their functional study, we use 0.75 as a relatively high threshold (hence in a ‘conservative’ sense) to define a manageable set of genes/isoforms for the validation study. However, it is desirable to link this threshold with a more statistically sound measure like p-value. As demonstrated in other differential analysis methods like DESeq, such a significance analysis is readily to be incorporated as a post-processing step. In future, we plan to add a follow-up step, statistical significance analysis, in our method so than a proper threshold using p-value can be used for selecting a final set of differential genes/transcripts.

Additionally, many genes identified by the three methods may also be associated with breast cancer. But our initial analysis shows that the pathways revealed by the large number of genes are diverse, hence hard to pin down their mechanistic involvement in the development of breast cancer recurrence. S1 Table lists the top ranked pathways obtained from Ingenuity Pathway Analysis, and S2 Table is the list of pathways obtained from KEGG pathway enrichment analysis (KEGG Pathway Database; <https://www.genome.jp/kegg/pathway.html>).

# S2. A validation study on MCF7 breast cancer cell line

From functional enrichment analysis using Ingenuity Pathway Analyses (IPA; Ingenuity Systems, http://www.ingenuity.com), the identified differentially expressed isoforms by our proposed method are significantly associated with several molecular and cellular functions. 223 isoforms are associated with proliferation of cells; 219 isoforms are associated with cell death; 157 isoforms are associated with migration of cells.

We performed a validation study of the three associated sets of isoforms on a time-course E2 induced MCF-7 breast cancer cell line RNAseq data ([GSE62789](http://www.ncbi.nlm.nih.gov/sites/entrez?db=gds&term=GSE62789%5bAccession%5d&cmd=search)). When generating the data, MCF-7 breast cancer cells were stimulated with estradiol (E2), and then mRNA concentration were collected using RNA-seq at 10 time points: 0, 5, 10, 20, 40, 80, 160, 320, 640, 1280 min. We downloaded the raw sequencing data and performed alignment using Tophat2 (Tophat v2.0.12). Cufflinks was utilized to estimate the RPKM of the isoforms.

We defined the activity of a set of isoforms from the correlation between the expression of the time-course samples and the time points. In specific, we first calculated the p-value (P-val) of rank correlation, and then converted into Z-score using

$$Z=\Phi^{-1}(1-\text{P-val})$$

where $\Phi(\cdot)$ is the CDF function of normal distribution. Finally, the mean value of Z-score of the set of isoforms was used as the measurement of the activity.

In the analysis, the null hypothesis is that the set of isoforms are randomly selected. Thus, for each set of identified isoforms, we randomly select 100,000 sets of isoforms of the same size, and calculate the activity, respectively, which approximates the null distribution. Finally, the p-value of the sets of identified isoforms is calculated by comparing its activity with the null distribution. As a result, the p-values of the three sets of isoforms associated with proliferation, cell death, and migration of cells are 0.043, 0.07 and 0.021, respectively, which shows that our identified sets of isoforms are active in the E2 induced MCF7 breast cancer cell line data.

# S3. Supplemental to Methods

In this paper, we have developed a Bayesian approach (BayesIso) that models both the between-sample variability and the within-sample variability, and combines the variability modeling with the differential state for differential analysis of isoforms. BayesIso can account for varying bias patterns along the isoforms. The distribution of differential state of the isoforms is estimated jointly with other model parameters, aiming to provide for an improved performance in detecting isoforms of less differentially expressed. In this section, we will give a more detailed mathematical description of the BayesIso approach as follows:

The conditional posterior distributions of the parameters $\mathbf{\beta}, \mathbf{\lambda}, \alpha, \alpha_{0}, \nu,$and the differential states $\mathbf{d}$ for the Gamma-Gamma model can be derived as follows:

The posterior probability of $\beta_{g,t,j}$ is defined by:

$P\left( \beta_{g,t,j_{1}} | \boldsymbol{y}_{g,j_{1}},U_{g,t,i}, \lambda_{g,t}^{\left( 1 \right)},\alpha\right)\sim P(\boldsymbol{y}_{g,j_{1}}|\beta_{g,t,j_{1}},U_{g,t,i})\times P(\beta_{g,t,j_{1}}| \lambda_{t,g}^{\left( 1 \right)},\alpha)$(S1)

$$\sim\prod_{i} \begin{aligned} \left( \left( \sum_{t} \left( s_{g,t,i}\beta_{g,t,j_{1}}x_{g,{i,j}_{1}}\exp\left( U_{g,t,i} \right) \right) \right)^{\boldsymbol{y}_{g,{i,j}_{1}}}\times\exp\left( -\sum_{t} \left( s_{g,t,i}\beta_{g,t,j_{1}}x_{g,{i,j}_{1}}\exp\left( U_{g,t,i} \right) \right) \right) \right) \\ \times\beta_{g,t,j_{1}}^{\alpha}\times\exp\left( -\lambda_{g,t}^{\left( 1 \right)}\beta_{g,t,j_{1}} \right), \end{aligned}$$

$P\left( \beta_{g,t,j_{2}} | \boldsymbol{y}_{g,j_{2}},U_{g,t,i}, \lambda_{g,t}^{\left( 2 \right)},\alpha\right)\sim P(\boldsymbol{y}_{g,j_{2}}|\beta_{g,t,j_{2}},U_{g,t,i})\times P(\beta_{g,t,j_{2}}| \lambda_{g,t}^{\left( 2 \right)},\alpha)$ (S2)

$$\sim\prod_{i} \begin{aligned} \left( \left( \sum_{t} \left( s_{g,t,i}\beta_{g,t,j_{2}}x_{g,{i,j}_{2}}\exp\left( U_{g,t,i} \right) \right) \right)^{\boldsymbol{y}_{g,{i,j}_{2}}}\times\exp\left( -\sum_{t} \left( s_{g,t,i}\beta_{g,t,j_{2}}x_{g,i,j_{2}}\exp\left( U_{g,t,i} \right) \right) \right) \right) \\ \times\beta_{g,t,j_{2}}^{\alpha}\times\exp\left( -\lambda_{g,t}^{\left( 2 \right)}\beta_{g,t,j_{2}} \right). \end{aligned}$$

$\lambda_{g,t}$ depends on differential state $d_{g,t}$. Thus, if $d_{g,t}=1$,

$\begin{matrix} \mathrm{P}\left(\lambda_{g,t}^{\left(1\right)}\left|\mathbf{\beta}_{g,t}^{\left(1\right)},\nu,\alpha,\alpha_{0}\right.\right)\sim\mathrm{P}\left(\mathbf{\beta}_{g,t}^{\left(1\right)}\left|\lambda_{g,t}^{\left(1\right)},\alpha\right.\right)\times\mathrm{P}\left(\lambda_{g,t}^{\left(1\right)}\left|\nu,\alpha_{0}\right.\right) \\ \sim Gamma\left(J_{1}\alpha+\alpha_{0},\sum_{j_{1}} \beta_{g,t,j_{1}}+\nu\right) \end{matrix}$, (S3)

$\begin{matrix} \mathrm{P}\left(\lambda_{g,t}^{\left(2\right)}\left|\mathbf{\beta}_{g,t}^{\left(2\right)},\nu,\alpha,\alpha_{0}\right.\right)\sim\mathrm{P}\left(\mathbf{\beta}_{g,t}^{\left(2\right)}\left|\lambda_{g,t}^{\left(2\right)},\alpha\right.\right)\times\mathrm{P}\left(\lambda_{g,t}^{\left(2\right)}\left|\nu,\alpha_{0}\right.\right) \\ \sim Gamma\left(J_{2}\alpha+\alpha_{0},\sum_{j_{2}} \beta_{g,t,j_{2}}+\nu\right) \end{matrix}$; (S4)

if $d_{g,t}=0$,

$\begin{matrix} \mathrm{P}\left(\lambda_{g,t}\left|\mathbf{\beta}_{g,t},\nu,\alpha,\alpha_{0}\right.\right)\sim\mathrm{P}\left(\mathbf{\beta}_{g,t}\left|\lambda_{g,t},\alpha\right.\right)\times\mathrm{P}\left(\lambda_{g,t}\left|\nu,\alpha_{0}\right.\right) \\ \sim Gamma\left(J\alpha+\alpha_{0},\sum_{j} \beta_{g,t,j}+\nu\right) \end{matrix}$. (S5)

The posterior distribution of $v$ is

$\begin{matrix} \mathrm{P}\left(v\left|\mathbf{\lambda},\mathbf{d},\alpha,\alpha_{0}\right.\right)\sim\mathrm{P}\left(\mathbf{\lambda}\left|\mathbf{d},v,\alpha,\alpha_{0}\right.\right)\times\mathrm{P}\left(v\right) \\ \sim Gamma\left(\left(\sum_{g,t} \left(1+d_{g,t}\right)\right)\alpha_{0}+a_{0},\sum_{g,t} \left(\lambda_{g,t}^{\left(1\right)}+\lambda_{g,t}^{\left(2\right)}\times d_{g,t}\right)+b_{0}\right). \end{matrix}$ (S6)

According to [[1](#_ENREF_1)], the posterior distribution of $\mathbf{d}$ given $\mathbf{\beta},\alpha,\alpha_{0}\text{ and }\nu$can be derived as:

$\begin{matrix} P\left(d_{g,t}\left|\mathbf{\beta},\lambda_{g,t},\alpha,\alpha_{0},\nu\right.\right)\sim P\left(\lambda_{g,t}\left|\mathbf{\beta},d_{g,t},\alpha,\alpha_{0},v\right.\right)P\left(d_{g,t}\right) \\ \sim\left(K_{1}K_{2}\frac{\left(\prod_{j} \beta_{g,t,j}\right)^{\alpha-1}}{\left(\nu+\sum_{j_{1}} \beta_{g,t,j_{1}}\right)^{J_{1}\alpha+\alpha_{0}}\left(\nu+\sum_{j_{2}} \beta_{g,t,j_{2}}\right)^{J_{2}\alpha+\alpha_{0}}}\right)^{d_{g,t}} \\ \text{ } \text{ }\times\left(K\frac{\left(\prod_{j} \beta_{g,t,j}\right)^{\alpha-1}}{\left(\nu+\sum_{j_{1}} \beta_{g,t,j_{1}}+\sum_{j_{2}} \beta_{g,t,j_{2}}\right)^{\left(J_{1}+J_{2}\right)\alpha+\alpha_{0}}}\right)^{1-d_{g,t}}\times\pi_{g,t} \end{matrix}$, (S7)

where

$K_{1}=\frac{\nu^{\alpha_{0}}\Gamma\left(J_{1}\alpha+\alpha_{0}\right)}{\Gamma^{J_{1}}\left(\alpha\right)\Gamma\left(\alpha_{0}\right)}$, $K_{2}=\frac{\nu^{\alpha_{0}}\Gamma\left(J_{2}\alpha+\alpha_{0}\right)}{\Gamma^{J_{2}}\left(\alpha\right)\Gamma\left(\alpha_{0}\right)}$, and $K=\frac{\nu^{\alpha_{0}}\Gamma\left(\left(J_{1}+J_{2}\right)\alpha+\alpha_{0}\right)}{\Gamma^{J_{1}+J_{2}}\left(\alpha\right)\Gamma\left(\alpha_{0}\right)}$.

$\pi_{g,t}$ is the prior of $d_{g,t}$ (differential state), which is assumed to be non-informative in our study, i.e., $\pi_{g,t}=0.5$.

The posterior distribution of $\alpha_{0}$and $\alpha$are given by:

$P\left(\alpha_{0}\left|\mathbf{\lambda},\mathbf{d},\alpha_{0},\nu\right.\right)\sim\prod_{g,t} \left(\frac{\nu^{\alpha_{0}}}{\Gamma\left(\alpha_{0}\right)}\left(\lambda_{g,t}^{\left(1\right)}\right)^{\alpha_{0}-1}\times\left(\frac{\nu^{\alpha_{0}}}{\Gamma\left(\alpha_{0}\right)}\left(\lambda_{g,t}^{\left(2\right)}\right)^{\alpha_{0}-1}\right)^{d_{g,t}}\right)$, (S8)

$\mathrm{P}\left(\alpha\left|\mathbf{\beta},\mathbf{\lambda}\right.\right)\sim\prod_{g,t} \left(\prod_{j_{1}} \frac{\left(\lambda_{g,t}^{\left(1\right)}\right)^{\alpha}}{\Gamma\left(\alpha\right)}\beta_{g,t,j_{1}}^{\alpha-1}\times\prod_{j_{2}} \frac{\left(\lambda_{g,t}^{\left(2\right)}\right)^{\alpha}}{\Gamma\left(\alpha\right)}\beta_{g,t,j_{2}}^{\alpha-1}\right)$. (S9)

When sample-level covariates (e.g., age, gender, treatment conditions, etc.) are available, a regression procedure will be used to obtain the corrected transcript expression level $\hat{\beta}_{t,g,j}$ as follows [[2](#_ENREF_2)]:

$\beta_{g,t,j}=\sum_{r}^{R} {w_{g,t,r}c}_{r}+\hat{\beta}_{g,t,j}+\varepsilon_{g,t}$ , (S10)

where $c_{r}(r=1, 2, \ldots, R)$is the sample-level covariate, $w_{g,t,r}(r=1, 2, \ldots, R)$ is the regression coefficient and $\varepsilon_{g,t}$ is an additive error term. Maximum likelihood estimation (MLE) for linear regression (or equivalent, least square fitting) can be used to estimate $w_{g,t,r}$, with which $\hat{\beta}_{g,t,j}$ can be obtained from the residual in fitting. The differential state (**d)** can then be estimated by sampling according to $P\left( d_{g,t} | \hat{\boldsymbol{\beta}},\lambda_{g,t},\alpha,\alpha_{0},\boldsymbol{v} \right)$, which needs to be updated (from Equation (S7)) as follows:

$$P\left( d_{g,t} | \hat{\boldsymbol{\beta}},\lambda_{g,t},\alpha,\alpha_{0},\boldsymbol{v} \right)=P\left( \lambda_{g,t} | \hat{\boldsymbol{\beta}},d_{g,t},\alpha,\alpha_{0},\boldsymbol{v} \right)P\left( d_{g,t} \right)$$

$\sim\left( K_{1}K_{2}\frac{{(\prod_{j} \hat{\beta}_{g,t,j})}^{\alpha-1}}{{(v+\sum_{j_{1}} \hat{\beta}_{g,t,j_{1}})}^{J_{1}\alpha+\alpha_{0}}{(v+\sum_{j_{2}} \hat{\beta}_{g,t,j_{2}})}^{J_{2}\alpha+\alpha_{0}}} \right)^{d_{g,t}}\times\left( K\frac{{(\prod_{j} \hat{\beta}_{g,t,j})}^{\alpha-1}}{{(v+\sum_{j_{1}} \hat{\beta}_{g,t,j_{1}}+\sum_{j_{2}} \hat{\beta}_{g,t,j_{2}})}^{{(J}_{1}+J_{2})\alpha+\alpha_{0}}} \right)^{{1-d}_{g,t}}\times\pi_{g,t}$. (S7’)

The main advantages of our proposed method, BayesIso, can be summarized as follows. First, it is a fully probabilistic approach that estimates the differential states and other model parameters iteratively. Assuredly accurate estimation of transcript expression is critical for differential state detection by comparing two groups of samples. At the same time, accurate detection of differential states benefits the estimation of transcript expressions, especially when the variance of transcript expressions among the samples (i.e., the between-sample variability) is high. Therefore, transcript expressions and differential states are tightly coupled when performing differential analysis of groups of samples. In the existing two-step approaches (e.g., Cuffdiff 2 and Ballgown), the detection of differentially expressed isoforms is carried out after expression estimation, not in a cooperative manner. BayesIso uses a joint model that combines the variability modeling of transcript expression with the differential state of transcripts for differential analysis of isoforms; the posterior probability of differential state is estimated jointly with other model parameters capturing expression variability through a MCMC sampling procedure.

Second, the differential state is embedded into the hierarchical model in a probabilistic way, in contrast to that conventional statistical tests calculate the significance of the difference between two groups of samples. Specifically in BayesIso, differential state is measured by the probability that the transcript expressions in two groups of samples come from two distributions rather than from the same distribution. The probabilistic formulation makes it possible to embed the differential state into the joint modeling of expressions of two groups of samples; the probability of differential state estimated jointly with other model parameters in turn provides an improved performance in detecting isoforms of less differentially expressed.

Third, the Poisson-Lognormal model in BayesIso provides a flexible way to model the various biases within each transcript. Cuffdiff 2 has tried to account for the bias along genomic location with a few known sources. Specific models are built for positional and sequence-specific biases, respectively. The parameters of the bias models are estimated in a global perspective, i.e., using all of the genes or a set of genes. However, the variability of real RNA-seq data may be much more complicated than that from known sources. The globally estimated parameters of the bias models are insufficient to account for the difference in biases among transcripts. In BayesIso, the variability of each exon/segment of each transcript is captured by one model parameter in the Poisson-Lognormal distribution, rather than estimated globally. Therefore, BayesIso models the within-sample variability specific to each transcript without relying on known sources.

# S4. Performance evaluation on abundance quantification

We evaluated the performance on abundance quantification with simulation datasets generated by varying parameter $\mathbf{U}$ in the Poisson-Lognormal model. Parameter $\mathbf{U}$ accounts for bias of RNAseq data along genomic location, resulting in the within-sample variability. We used the average correlation of the estimated expression and the ground truth transcript expression across multiple samples to assess the accuracy of abundance quantification. We compared the abundance (β in Eq. (3) as defined in the Methods section) estimated by our method with two measurements of abundance estimated by Cufflinks and Cuffdiff 2: ‘reads-per-kilobase-per-million’ (RPKM) and ‘external_normalized_count’ (ENC). RPKM [[3](#_ENREF_3)] is calculated by normalizing the reads by length of genomic features (e.g., genes/isoforms/exons) and the total number of mapped reads. ENC is an alternative measurement estimated by Cuffdiff 2 to take into account the phenotype information and variance of the samples.

First, we varied parameter $\tau$ that affects the overall within-sample variability, with other model parameters set as $\alpha=1$, $\alpha_{0}=0.5$, and $v=0.1$. In general, the smaller the precision parameter $\tau$, the higher the overall within-sample variability. From S6(a) Fig, we can see that, at different $\tau$, the average correlations of our estimation were consistently higher than those of RPKM and ENC. Note that although RPKM and ENC were calculated by different methods, their correlations to the true expression level across samples were about the same.

Second, we tested the performance on simulation data with different bias patterns along genomic location. Rather than randomly drawn from $N(0,\tau)$, parameter $\mathbf{U}$ is designed to follow different bias patterns for different sets of genes. We simulated four patterns, as shown by the blue curves in S6(b) Fig. S6, to mimic the observed patterns from real RNA-seq data in our studies. We can see from the estimated patterns shown in S6(b) Fig (the red curves) that our proposed model is able to capture the bias patterns accurately. The correct estimation of the bias pattern consequently contributes to more accurate abundance estimation (S6(a) Fig). As shown by the bar plot in S6(b) Fig, the performances on groups of genes with different bias patterns are comparable, indicating that our model can deal with various bias patterns.

# S5. Performance evaluation on identification of differential isoforms

**Overview of the simulation study**. We generated simulation data on 697 genes with 1394 isoforms, where 666 isoforms were randomly assigned as differentially expressed yet 728 isoforms as non-differentially expressed. We calculated signal-to-noise-ratio (SNR) of each truly differentially expressed isoform from the simulation data, and then divided them into three groups listed in S3 Table. The histogram of the SNR of all truly differentially expressed isoforms is shown in S7 Fig.

We applied the three competing methods (BayesIso, Cuffdiff 2 and Ballgown) to the simulated data, and the experimental results are shown in S8 Fig. In our Bayesian approach, the posterior probability of the differential states of the isoforms is estimated, while Cuffdiff 2 and Ballgown calculate the p-value from statistic tests. ‘Prob(d=1)>0.75’ (for BayesIso) or ‘p-value<0.05’ (for other competing methods) is used as the criterion for differential isoform identification. S9 Fig shows the performance comparison on abundance quantification in which the groups of isoforms are grouped according to their SNRs. We can see from the figure that our proposed method outperforms the other two methods with a robust performance when SNR of the differential isoforms decreased.

**Performance comparison with varying model parameters.** We varied parameter $\alpha$ and $\alpha_{0}$ in the Gamma-Gamma model to evaluate the performance on differentially expressed isoform identification, as compared with two existing methods: Cuffdiff 2 and Ballgown. When varying $\alpha$, we set $\alpha_{0}=0.5$, $v=0.1$, $\tau=1.78$; when varying $\alpha_{0}$, we set $\alpha=1.5$, $v=0.1$, $\tau=1.78$. Precision, recall and F-score are used to evaluate the performance. F-score is calculated by the following definition:

$\text{F-score = }\frac{2\times\text{precision}\times\text{recall}}{\text{precision}+\text{recall}}$.

S4 Table lists the results of performance comparison on varying parameter settings. The criteria of P-val<0.05 (for Cuffdiff 2 and Ballgown) and prob(d)>0.75 (for BayesIso) are used, respectively, for identifying differentially expressed isoforms. As we can see, our method consistently outperforms the other methods on simulation data generated with various parameters. Cuffdiff 2 achieves a high precision; however, it misses many differentially expressed isoforms. Ballgown can identify more differentially expressed isoforms but with less precision. From the simulation study, it is evident that parameter $\alpha$ affects the expression level of the isoforms. In general, as $\alpha$ increases, so does the average expression level of the isoforms. More importantly, parameter $\alpha_{0}$ influences the difference of abundance comparing samples from two phenotypes taking into account the variance. For larger $\alpha_{0}$ values, the difference between two phenotypes becomes smaller. From the tables we can see that our method is more effective when the isoform abundance is generally lower, or the isoforms are less differentially expressed. The improved performance of BayesIso in differential analysis is gained from: (1) using a model to account for both within-sample variability and between-sample variability; (2) estimating differential states of isoforms by a Bayesian method using a joint model of the variability and differential state.

We can also use signal-to-noise-ratio (SNR) as a measure for the differential level of isoforms, calculated as follows, to show the advantage of BayesIso in detecting isoforms of less differentially expressed:

$SNR=10\log_{10} \left( \frac{\left| mean\left( cond1 \right)-mean\left( cond2 \right) \right|}{sqrt\left( var\left( cond1 \right)+var\left( cond2 \right) \right)} \right)$.

Ground truth differentially expressed isoforms are divided into three groups according to SNR. Performances on different groups of truly differential isoforms and non-differential isoforms are evaluated seperately, as shown in S5 Table. When the performance on all of the isoforms is compared, the recall performance of Cuffdiff 2 is the lowest, which affects its overall performance measured by F-score. According to the performance on different groups of isoforms, we can see that BayesIso achieves a much higher recall when the isoforms are less differentially expresed (‘diff-group2’ with -3 dB< SNR <-1 dB). This improvement can be attributed in part to the more accurate abundance quantification of BayesIso (as shown S6 Fig). We can also see that the false positive rate (defined by the portion of non-differential isoforms included) of our method is lower than Ballgown. Note that the false positive rate of Cuffdiff 2 is low because fewer differential isoforms are identified.

We also evaluated the performance of the competing methods on genes with 3, 4, and 5 isoforms. The experimental results demonstrates that our proposed method outperforms the other methods in multiple scenarios (S6 Table). The performance comparison result is consistent with experiments on genes with two isoforms. Additionally, the performance on genes with a single isoform is also listed in the last row of S6 Table.

# S6. Simulation study using RNAseqReadSimualtor

We also generated synthetic data using another RNA-seq simulator (RNAseqReadSimulator [[4](#_ENREF_4)]) to test the performance of the competing methods. 1,000 isoforms from 500 genes are randomly selected for the experiment, where 498 isoforms are differentially expressed.

When simulating the RNA-seq data, we randomly select isoforms to follow four different bias patterns along the genomic location as shown in S10(a) Fig. In specific, isoforms are divided into 4 groups, and each group including around 250 isoforms, followed a specific bias pattern. S10(b) Fig shows the bias patterns inferred by our approach, which indicates that our proposed method can capture varying bias patterns by estimating parameter **U**. Note that RNAseqReadSimulator simulates the bias patterns but not the parameter **U**. Thus, there is no ground truth for parameter **U**. However, the similarity between the estimated patterns and the simulated patterns demonstrates the capability of BayesIso in capturing varying bias patterns.

To evaluate the performance on the detection of differentially expressed isoforms, we also looked into subgroups of isoforms with different SNR in addition to the overall performance on all of the isoforms. Similar to the study in Section S5, we calculate signal-to-noise-ratio (SNR) of each truly differentially expressed isoform from the simulation data, and then divide them into three groups as listed in S7 Table. The histogram of the SNR of all truly differentially expressed isoforms is shown in S11 Fig. S12 Fig shows the histograms of the posterior probability of differential state estimated by our method, the p-value calculated from Cuffdiff 2 and Ballgown. With ‘prob(d=1) >0.75’ for our method (BayesIso) and ‘p-value<0.05’ for the other two methods as the criterion for differential expression, the precision, recall, and F-score of the competing methods are listed in S8 Table. Consistent with previous comparison, our method achieves the highest overall performance measured by F-score. Cuffdiff2 achieves very high precision, yet the recall is very low. The BayesIso method achieves much higher detection power (much higher recall) on the moderately differential isoforms (-3dB<SNR<-1dB).

# S7. Performance Comparison on Real RNA-seq Data

We used two benchmark datasets to evaluate the performance of the competing methods on differential analysis of RNA-seq data. Both datasets are part of the MicroArray Quality Control Project (MAQC) project for benchmarking microarray technology [[5](#_ENREF_5), [6](#_ENREF_6)] as well as to characterize RNA-seq technology, which includes replicated samples of human brain reference RNA (hbr) and universal human reference RNA (uhr). The first data set is the Sequencing Quality Control (SEQC) dataset, which includes RNA spike-in controls along with the true RNA library. The RNA spike-in controls are a set of synthetic RNAs from External RNA Control Consortium (ERCC), which can be used as the benchmark. The second data set is another set of samples of hbr and ubr. The expression of close to 1000 genes in uhr and hbr were validated by TaqMan qRT-PCR, which can be used as the benchmark.

**Performance comparison benchmarked by spike-in ERCC RNAs**

We first compared the performance of BayesIso with the four existing methods (DESeq, edgeR, DSS, EBSeq and SGNB [[7](#_ENREF_7)]) on the Sequencing Quality Control (SEQC) dataset with ERCC spike-in controls [[8](#_ENREF_8)]. 92 artificial transcripts are mixed into a real RNA-Seq library with different ratios (1:1 for none differentially expressed genes, and 4:1, 2:3 and 1:2 for differentially expressed genes), which are used as ground truth for differential analysis. Gene level counts are downloaded from http://bitbucket.org/soccin/seqc, with 5 replicates in each group. S13 Fig shows the ROC curves of the five competing methods. We can see that BayesIso shows the best performance among all five methods by achieving an AUC very close to 0.97. The second best method is SGNB with an AUC about 0.88; the third best method is DSS with an AUC about 0.85. DESeq (AUC=0.7624) and edgeR (AUC=0.7675) are of very close performance, which is consistent with the previous results reported by Rapaport *et al.* [[8](#_ENREF_8)]. EBSeq, on the other hand, has the least favorable performance (AUC=0.71) on this specific dataset and it fails to detect the most strongly differentially expressed genes: its sensitivity is less than 0.1 when its specificity is about 0.9. By a close examination of the ‘left’ ROC curves of the five methods, we can further infer that BayesIso exhibits significantly better precision than the competing methods, whose sensitivity goes up to 0.7 before any degradation in specificity.

**Performance comparison benchmarked by TaqMan data**

The set of roughly 1000 genes measured by qRT-PCR in the TaqMan data is a set of more comprehensive benchmarks as they span a wider range of expression ratios and represents a sampling of true human transcripts [[9](#_ENREF_9)]. We evaluated the ability of all of the competing methods to detect differentially expressed genes on another RNA-seq data set with replicated samples of hbr and ubr. These samples were generated by Dr. Dudoit from University of California at Berkeley [[10](#_ENREF_10)] from NCBI sequence read archive (SRA) with ID SRX016359 and SRX016367, with 7 samples in each phenotype. To apply all of the competing methods on this data set for differential analysis, we first performed alignment using ‘TopHat 2 (TopHat v2.0.12)’ with UCSC hg19 as the reference sequence. Then, cuffdiff 2 and Ballgown can be applied on the aligned reads for differential analysis on the RefSeq genes. For other count-based methods, i.e., DESeq, edgeR, EBSeq, and DSS, the required input, read count, is calculated from RPKM that is estimated by cufflinks for fair comparison. All of the methods are run under their default setting, and q-value < 0.05 is used as the criteria for differential genes for cuffdiff 2, Ballgown, DESeq, edgeR, and DSS, while Prob(DE) > 0.95 is used for BayesIso and EBSeq.

TaqMan qRT-PCR measurements of roughly 1000 genes were downloaded from https://bitbucket.org/soccin/seqc. Among the roughly 1000 genes, 844 genes are overlapped with RefSeq genes, which are used to benchmark the performance on differential analysis. We use the absolute log2 ratio of fold change (| log_2_FC |) of the expression between the two phenotypes to determine differentially expressed genes and non-differentially expressed genes. Genes with | log_2_FC | larger than a threshold $T$ are considered as differentially expressed, while the other genes are non-differentially expressed. We vary the threshold T from 2.5 to 1 (5.6 × expression change to 1.5 × expression change measured by qRT-PCR) to evaluate the performance of the competing methods at decreasing cutoff values of qRT-PCR expression change, which defines sets of differentially expressed genes with decreasing differential level. We use Recall, Precision, and F-score as the metrics, as shown in S14 Fig. From S14(a) Fig, we can see that the overall performance, evaluated by F-score, of BayesIso outperforms all other methods in various scenarios. Moreover, F-score increases as the differential level decreases, which indicates the ability of BayesIso in detecting less differentially expressed genes. As shown in S14(b) Fig, the recall of EBSeq is higher than BayesIso; however, the precision of EBSeq is much lower, as too many genes are detected as differentially expressed. The precisions of DSS and Ballgown are higher than BayesIso, as they detect much fewer differentially expressed genes, which can be inferred by their poor performance in recall. Therefore, the superiority of BayesIso over the competing methods has been demonstrated on genes with different differential levels.

**Summary of performance evaluation.** We have compared the performance of BayesIso with Cuffdiff 2 and Ballgown using extensive studies. BayesIso consistently outperforms the other methods in all of the scenarios studied, with F-score as the metric to evaluate the overall performance in differential isoform identification. Cuffdiff 2 is very conservative as it can detect a few differentially expressed isoforms at high precision but missing a lot of others. Ballgown can detect more differential isoforms at the cost of precision. BayesIso has significantly increased the performance in detecting true differential isoforms as evaluated by recall, and with little compromise in precision. From further experiments on isoforms with various differential levels, BayesIso has been demonstrated to be more effective in detecting isoforms of less differentially expressed. The improved performance is gained from iteratively estimating the differential state and the parameters of the joint modeling of both types of expression variability. At the same time, the various biases of the transcripts are well captured, leading to more accurate estimation of transcript expression. Overall, the hierarchical Bayesian framework contributes to the improved performance of BayesIso.

# References

1. Wei Z, Li H. A Markov random field model for network-based analysis of genomic data. Bioinformatics. 2007;23(12):1537-44. Epub 2007/05/08. doi: 10.1093/bioinformatics/btm129. PubMed PMID: 17483504.

2. Ritchie ME, Phipson B, Wu D, Hu Y, Law CW, Shi W, et al. limma powers differential expression analyses for RNA-sequencing and microarray studies. Nucleic Acids Res. 2015;43(7):e47. Epub 20150120. doi: 10.1093/nar/gkv007. PubMed PMID: 25605792; PubMed Central PMCID: PMCPMC4402510.

3. Trapnell C, Williams BA, Pertea G, Mortazavi A, Kwan G, van Baren MJ, et al. Transcript assembly and quantification by RNA-Seq reveals unannotated transcripts and isoform switching during cell differentiation. Nature biotechnology. 2010;28(5):511-5. Epub 2010/05/04. doi: 10.1038/nbt.1621. PubMed PMID: 20436464; PubMed Central PMCID: PMC3146043.

4. Li W, Jiang T. Transcriptome assembly and isoform expression level estimation from biased RNA-Seq reads. Bioinformatics. 2012;28(22):2914-21. Epub 2012/10/13. doi: 10.1093/bioinformatics/bts559. PubMed PMID: 23060617; PubMed Central PMCID: PMC3496342.

5. Shi L, Campbell G, Jones WD, Campagne F, Wen Z, Walker SJ, et al. The MicroArray Quality Control (MAQC)-II study of common practices for the development and validation of microarray-based predictive models. Nature biotechnology. 2010;28(8):827-38. Epub 2010/08/03. doi: 10.1038/nbt.1665. PubMed PMID: 20676074; PubMed Central PMCID: PMC3315840.

6. Shi L, Reid LH, Jones WD, Shippy R, Warrington JA, Baker SC, et al. The MicroArray Quality Control (MAQC) project shows inter- and intraplatform reproducibility of gene expression measurements. Nature biotechnology. 2006;24(9):1151-61. Epub 2006/09/12. doi: 10.1038/nbt1239. PubMed PMID: 16964229; PubMed Central PMCID: PMC3272078.

7. Liu Y, Wang J, Wu S, Yang J. A model for isoform-level differential expression analysis using RNA-seq data without pre-specifying isoform structure. PLoS One. 2022;17(5):e0266162. Epub 20220516. doi: 10.1371/journal.pone.0266162. PubMed PMID: 35576204; PubMed Central PMCID: PMCPMC9109925.

8. Rapaport F, Khanin R, Liang Y, Pirun M, Krek A, Zumbo P, et al. Comprehensive evaluation of differential gene expression analysis methods for RNA-seq data. Genome Biol. 2013;14(9):R95. Epub 2013/09/12. doi: gb-2013-14-9-r95 [pii]

10.1186/gb-2013-14-9-r95. PubMed PMID: 24020486.

9. Canales RD, Luo Y, Willey JC, Austermiller B, Barbacioru CC, Boysen C, et al. Evaluation of DNA microarray results with quantitative gene expression platforms. Nature biotechnology. 2006;24(9):1115-22. Epub 2006/09/12. doi: 10.1038/nbt1236. PubMed PMID: 16964225.

10. Bullard JH, Purdom E, Hansen KD, Dudoit S. Evaluation of statistical methods for normalization and differential expression in mRNA-Seq experiments. BMC bioinformatics. 2010;11:94. Epub 2010/02/20. doi: 10.1186/1471-2105-11-94. PubMed PMID: 20167110; PubMed Central PMCID: PMC2838869.
