# Supplementary figures and images for: Bayesian identification of differentially expressed isoforms using a novel joint model of RNA-seq data
Source: PLoS Comput Biol. 2025 Jan 31;21(1):e1012750. doi: 10.1371/journal.pcbi.1012750 (PMC11819608; doi:10.1371/journal.pcbi.1012750)

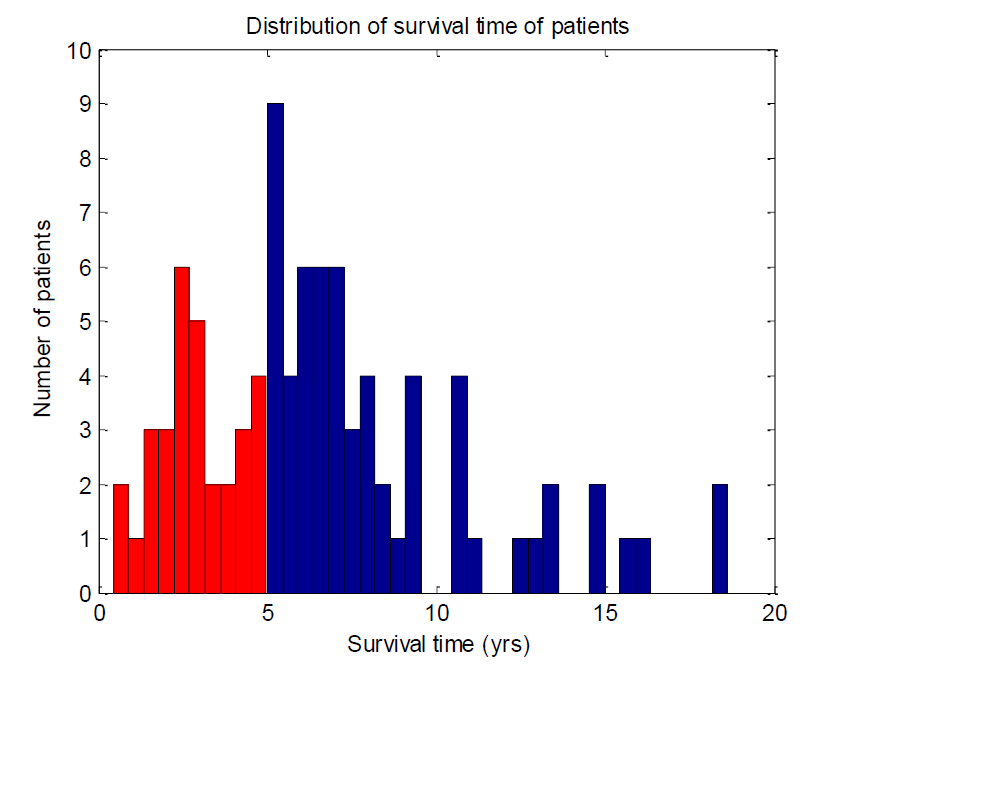

Supplement: S1 Fig — (TIF) [file pcbi.1012750.s002.tif]

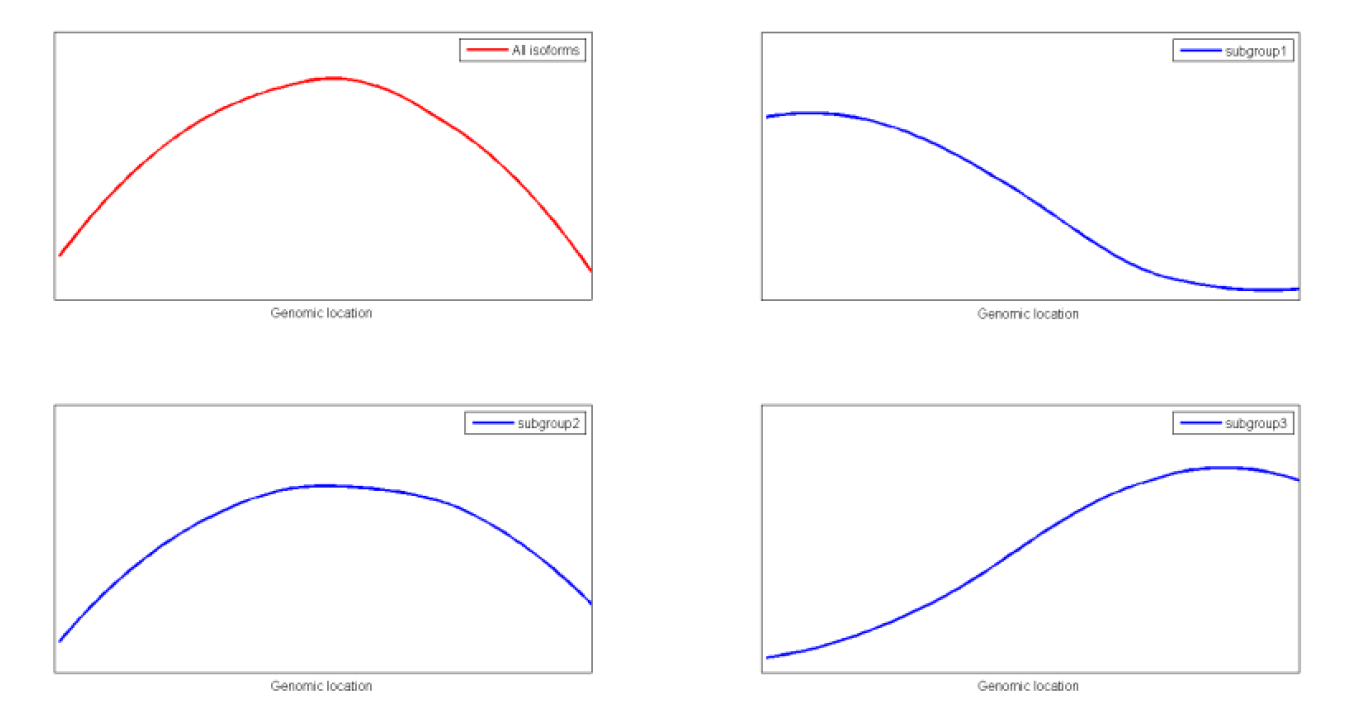

Supplement: S2 Fig — The mean bias pattern of all the isoforms is shown by the red curve in the up-left figure. However, different sets of isoforms exhibit varying bias patterns. The three blue curves show the mean bias patterns of different groups of isoforms. The isoforms are grouped according to their bias patterns. (TIF) [file pcbi.1012750.s003.tif]

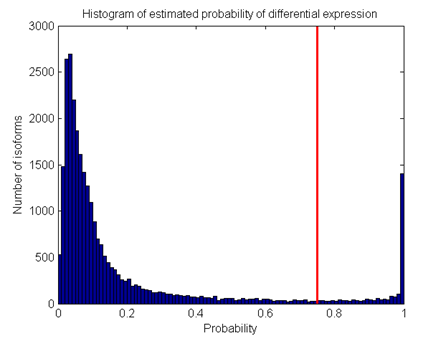

Supplement: S3 Fig — Red line denotes Prob(d=1) = 0.75. (TIF) [file pcbi.1012750.s004.tif]

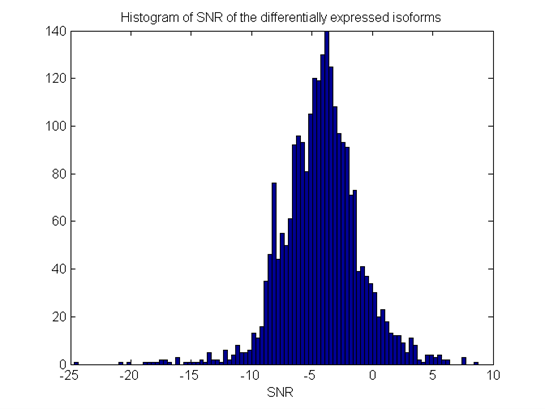

Supplement: S4 Fig — (TIF) [file pcbi.1012750.s005.tif]

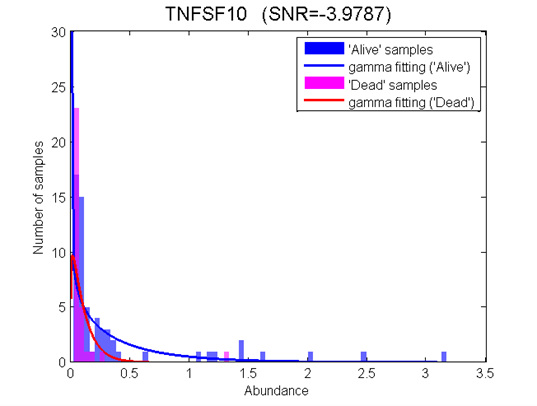

Supplement: S5 Fig — Blue bars denote the abundance in the ‘Alive’ group, and the blue curve denotes the fitting of the blue bars with a gamma distribution. Red bars denote the abundance in the ‘Dead’ group, and the red curve denotes the fitting of the red bars with a gamma distribution. (TIF) [file pcbi.1012750.s006.tif]

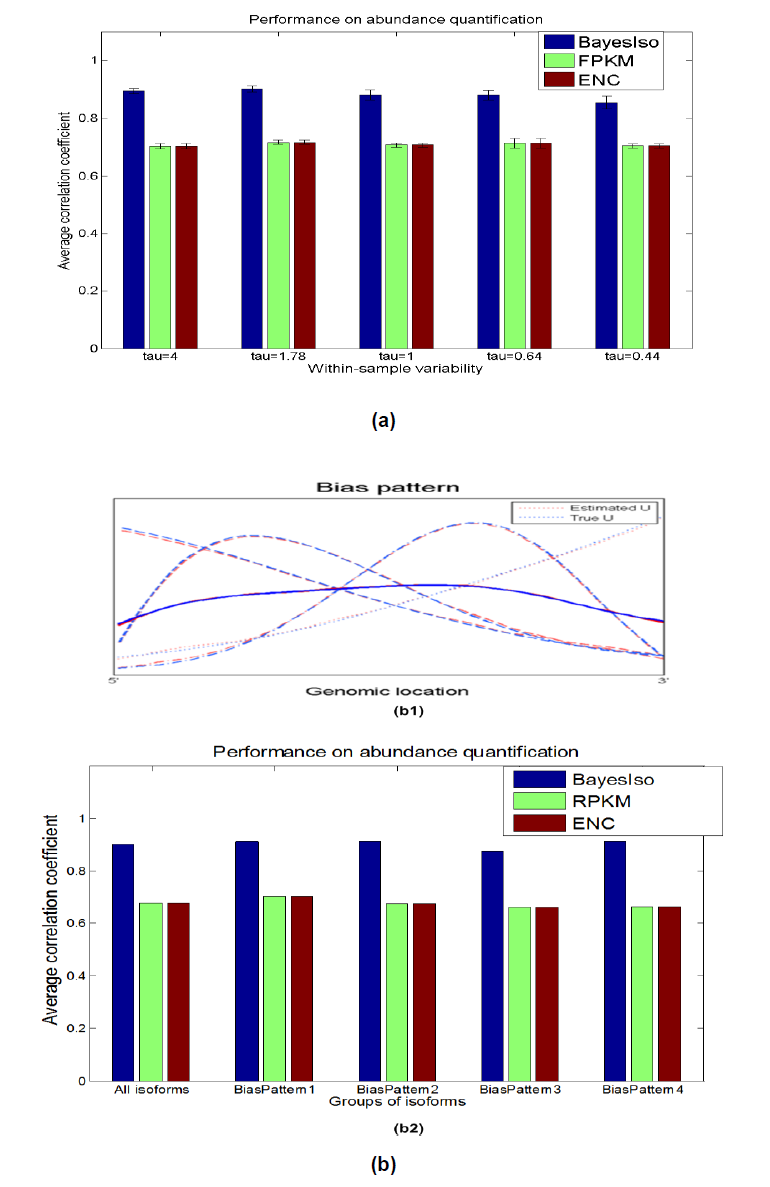

Supplement: S6 Fig — (a) Different overall within-sample variability; (b) different bias patterns along the genomic location. Average correlation coefficient between the estimated abundance and the true abundance of the isoforms is used to evaluate the performance. (TIF) [file pcbi.1012750.s007.tif]

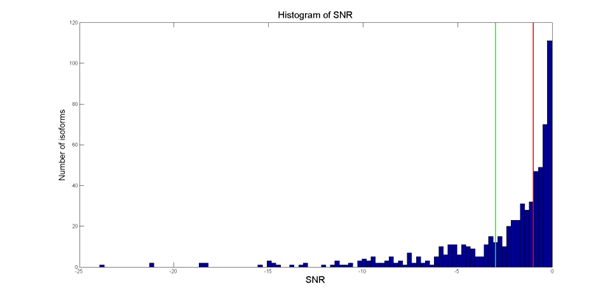

Supplement: S7 Fig — Red line denotes SNR = −1 dB; green line denotes SNR = −3 dB. (TIF) [file pcbi.1012750.s008.tif]

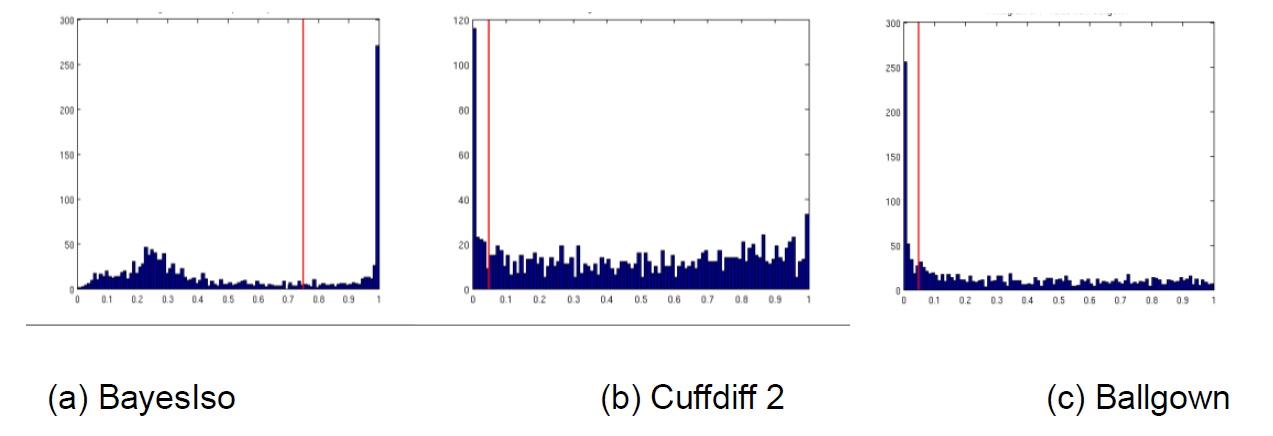

Supplement: S8 Fig — (TIF) [file pcbi.1012750.s009.tif]

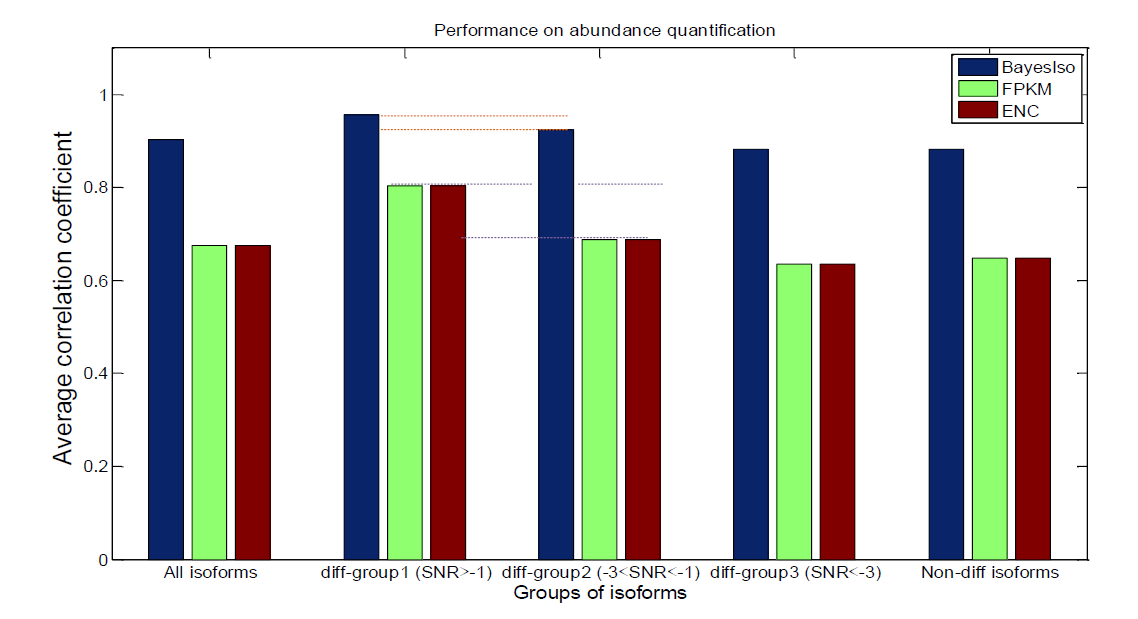

Supplement: S9 Fig — The average correlation coefficients of all of the isoforms of the three competing methods are listed by the left three bars, while the performance on the three groups of differentially expressed isoforms and the non-differential isoforms are shown by the other 4 groups of bars. (TIF) [file pcbi.1012750.s010.tif]

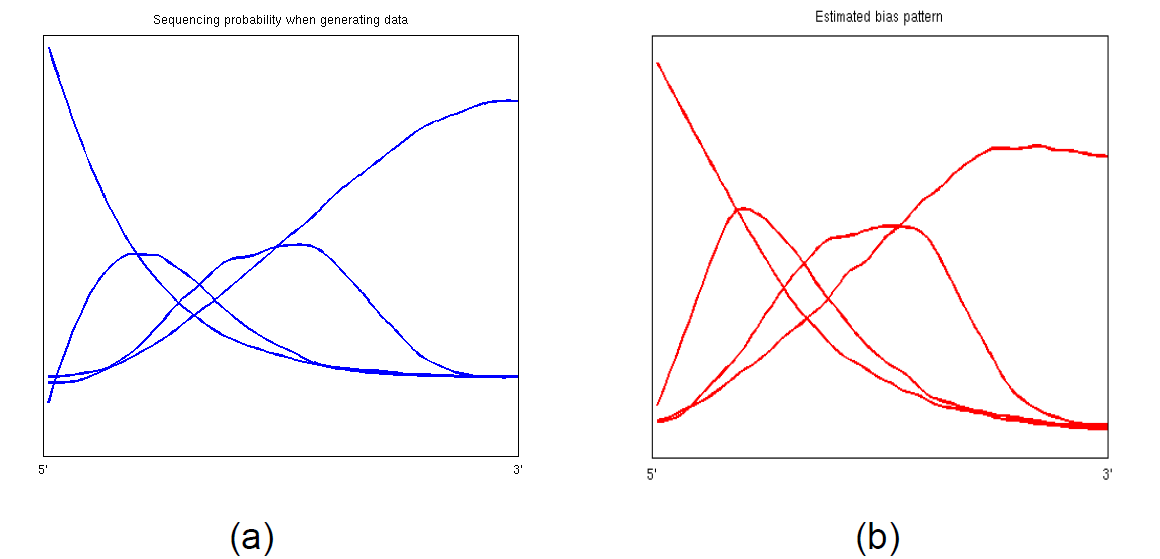

Supplement: S10 Fig — (a) Four different bias patterns presented by the curves are simulated by varying the sequencing probabilities according to genomic location. (b) Estimated biased patterns indicated by expU of the four groups of isoforms. (TIF) [file pcbi.1012750.s011.tif]

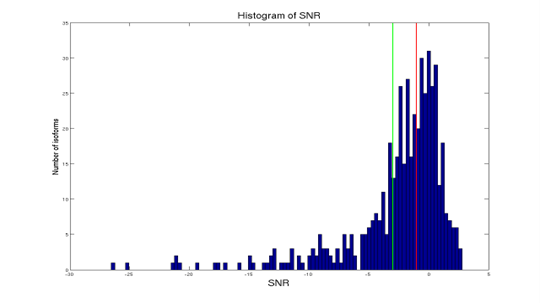

Supplement: S11 Fig — (TIF) [file pcbi.1012750.s012.tif]

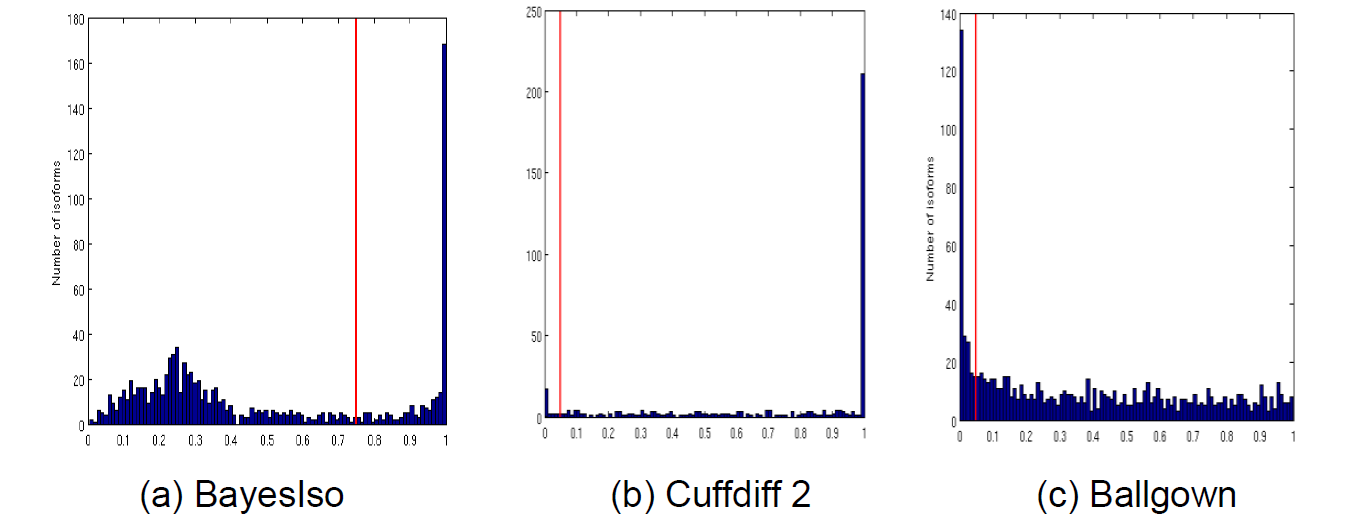

Supplement: S12 Fig — (TIF) [file pcbi.1012750.s013.tif]

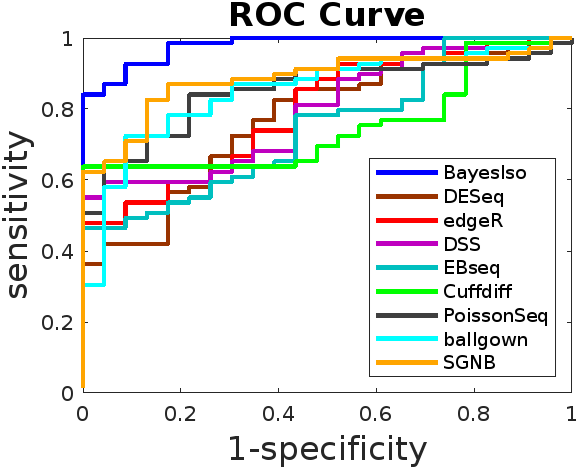

Supplement: S13 Fig — (TIF) [file pcbi.1012750.s014.tif]

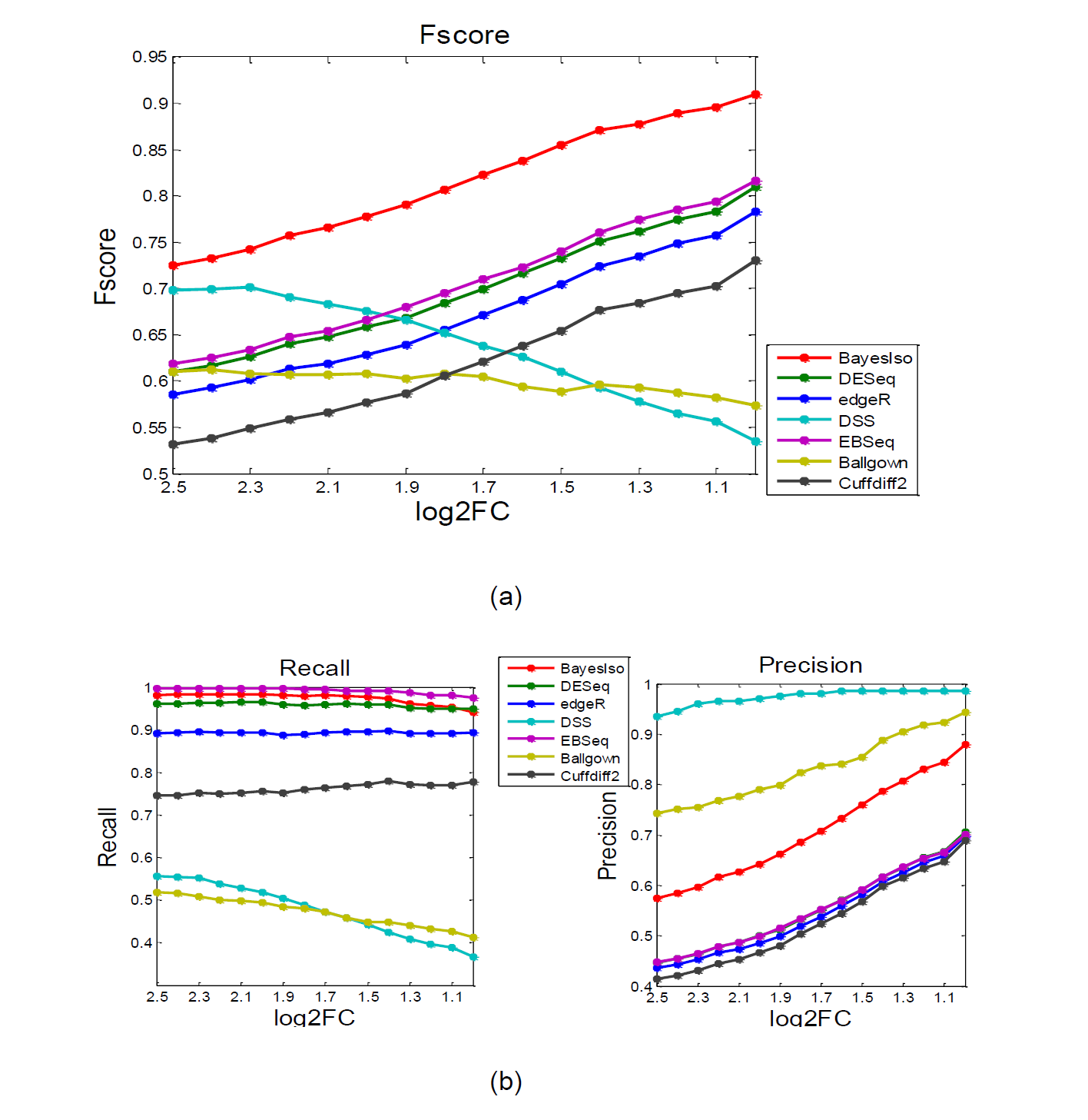

Supplement: S14 Fig — (TIF) [file pcbi.1012750.s015.tif]
